# IFNγ blockade in *Mycobacterium tuberculosis* infected macaques alters the granuloma environment but not bacterial control

**DOI:** 10.1038/s41467-026-72421-9

**Published:** 2026-04-25

**Authors:** Shunsuke Sakai, Sivaranjani Namasivayam, Keith D. Kauffman, Eduardo Fukutani, Artur T. L. Queiroz, Christine E. Nelson, Erin F. McCaffrey, Ploenchan Chetchotisakd, Jay Buchanan, Alan Sher, Carl G. Feng, Joel D. Ernst, Steven M. Holland, Katrin D. Mayer-Barber, Bruno B. Andrade, Christa S. Zerbe, Laura E. Via, Daniel L. Barber

**Affiliations:** 1 T Lymphocyte Biology Section, Laboratory of Parasitic Diseases, National Institute of Allergy and Infectious Diseases, National Institutes of Health, Bethesda, MD USA; 2 Immunobiology Section, Laboratory of Parasitic Diseases, National Institute of Allergy and Infectious Diseases, National Institutes of Health, Bethesda, MD USA; 3 Laboratório de Pesquisa Clínica e Translacional, Instituto Gonçalo Moniz, Fundação Oswaldo Cruz, Salvador, Brazil; 4 Spatial Immunology Unit, Laboratory of Parasitic Diseases, National Institute of Allergy and Infectious Diseases, National Institutes of Health, Bethesda, MD USA; 5 Division of Infectious Diseases and Tropical Medicine, Srinagarind Hospital, Khon Kaen University, Khon Kaen, Thailand; 6 Immunology and Host Defense Group, Faculty of Medicine and Health, The University of Sydney, Sydney, New South Wales Australia; 7 Division of Experimental Medicine, Department of Medicine, University of California, San Francisco, CA USA; 8 Immunopathogenesis Section, Laboratory of Clinical Immunology and Microbiology, National Institute of Allergy and Infectious Diseases, National Institutes of Health, Bethesda, MD USA; 9 Inflammation and Innate Immunity Section, Laboratory of Clinical Immunology and Microbiology, National Institute of Allergy and Infectious Diseases, National Institutes of Health, Bethesda, MD USA; 10 Division of Infectious Diseases, John Hopkins School of Medicine, Baltimore, MD USA; 11 Department of International Health, Bloomberg School of Public Health, Johns Hopkins University, Baltimore, MD USA; 12 Infectious Disease Fellowship Program, Laboratory of Clinical Immunology and Microbiology, National Institute of Allergy and Infectious Diseases, National Institutes of Health, Bethesda, MD USA; 13 Tuberculosis Research Section, Laboratory of Clinical Immunology and Microbiology, Division of Intramural Research, National Institute of Allergy and Infectious Disease, National Institutes of Health, Bethesda, MD USA; 14 Tuberculosis Imaging Program, Division of Intramural Research, National Institute of Allergy and Infectious Disease, National Institutes of Health, Bethesda, MD USA

**Keywords:** Tuberculosis, Infection, Pathogens

## Abstract

IFNγ is considered the primary mediator of adaptive immunity to *Mycobacterium tuberculosis* (Mtb) infection. In mice, control of Mtb requires IFNγ. In humans, IFNγ is critical for resistance to infection with non-tuberculous mycobacteria (NTM), but its relative requirement for control of pulmonary tuberculosis (TB) is less clear. Here we block IFNγR1 signaling in macaques at different times following Mtb infection. IFNγ blockade from day 45 to 49 post-infection rapidly reduced ^18^FDG-PET/CT scores and broadly enhanced anti-viral-like inflammatory responses. Strikingly, IFNγR1 blockade for the first three months of infection had no impact on bacterial loads despite suppression of bioactive IFNγ in granulomas and changes to host immune responses and granuloma structure. We find individuals from Mtb endemic regions with anti-IFNγ neutralizing autoantibodies who develop NTM disease and not TB despite evidence of previous exposure to Mtb. Lastly, we show that mice over-estimate the importance IFNγ in host resistance to TB due to a species-specific induction of iNOS by IFNγ. Thus, while IFNγ has immunoregulatory effects in granulomas, normal resistance to TB in macaques and humans likely requires little IFNγR1 signaling during infection, indicating that the major mechanisms of adaptive immunity to Mtb infection remain unknown.

## Abstract (web summary)

Here, the authors show that IFNγ blockade does not impair resistance to *Mycobacterium tuberculosis* infection in macaques, opposed to what has been observed in mice, due to a species-specific induction of iNOS by IFNγ.

## Introduction

M*ycobacterium tuberculosis* (Mtb) infection is one of the leading causes of global mortality. The only available vaccine for tuberculosis (TB), Bacillus Calmette-Guerin (BCG) protects infants against severe forms of TB but does little to prevent disease in adolescents and adults. A highly effective TB vaccine would have a tremendous beneficial impact on global public health. However, due to the lack of robust correlates of protection, TB vaccine development is largely empirical, which contributes to the slow rate of conceptual and practical progress. A better understanding of the mechanisms of protection could help guide the rational design of TB vaccine candidates.

CD4 T cells are particularly critical in control of Mtb infection and are likely to be a major contributor to vaccine-elicited protection. The anti-mycobacterial effect of CD4 T cells is largely attributed to their production of interferon-gamma (IFNγ), as mice deficient in IFNγ^1–3^ or CD4 T cells^4–7^ are highly susceptible to mycobacterial infection. In Mtb-infected mice, CD4 T cell-derived IFNγ acts, at least in part, on infected macrophages (Macs), as Macs lacking MHCII^8^ or IFNγ receptor (IFNγR)^9^ are unable to suppress growth of Mtb. The role of IFNγ in driving nitric oxide synthase 2 (NOS2) expression has been thought to be essential for the protective effects of IFNγ during Mtb infection^10–14^. Thus, data from mice has led to the model where CD4 T cells secrete IFNγ onto Mtb-infected Macs to drive NOS2, which generates reactive free radicals that exert anti-mycobacterial effects as well as control detrimental inflammation^9^. However, IFNγ does not drive the induction of NOS2 in human macrophages as it does in murine cells^15–19^, and the role of IFNγ at the site of infection in human lungs is poorly understood.

It is also clear that CD4 T cells in mice can mediate control of Mtb infection independent of IFNγ production^4,5,20,21^. Our previous studies indicated that CD4 T cell-derived IFNγ is critical in limiting extrapulmonary growth of the bacteria, while IFNγ-independent pathways have a large contribution to T cell-mediated suppression of Mtb growth in the lungs^22^. Children with inborn errors in *IFNG, IFNGR1, IFNGR2*, or the downstream signaling molecules *STAT1 and IRF1*, are susceptible to mycobacterial infection (known as Mendelian susceptibility to mycobacterial diseases [MSMD]), however these individuals much more frequently manifest with extrapulmonary non-tuberculous mycobacterial (NTM) infections as compared to TB^23^. Similarly, adults that develope neutralizing autoantibodies (autoAbs) against IFNγ are also highly susceptible to mycobacterial infections, however, they also more often develop extrapulmonary NTM infections rather than typical pulmonary TB^24,25^.

Additional insight into the role of IFNγ in Mtb infection has come from the study of “resisters”, i.e., individuals that have been highly exposed to contagious TB index cases but persistently test negative in the IFNγ release assay (IGRA) blood test. It was found that these individuals are infected with Mtb, but their antigen (Ag)-specific CD4 T cells produce little IFNγ^26,27^. Instead, their Mtb-specific T cells are skewed towards a Th17 and Treg phenotype^28^. Thus, some individuals are able to contain Mtb infection despite the fact that their Mtb-specific CD4 T cells fail to polarize into IFNγ-secreting Th1 cells. Collectively, these observations suggest there are IFNγ-independent mechanisms of CD4 T cell-mediated control of Mtb infection, but their relative importance is not clear. Here we block IFNγ signaling in Mtb-infected rhesus macaques using a soluble decoy receptor to examine the role of IFNγ within granulomas. We also examine Mtb-specific T cell responses in NTM patients with neutralizing anti-IFNγ autoAbs to determine if they had ever been exposed to Mtb.

## Results

### Short-term IFNγ blockade after TB granuloma formation in macaques

We first sought to determine the how IFNγR signaling regulates the composition and function of cells in granulomas. In designing the experiment, we had to contend with several potential caveats. First, if bacterial loads increased in the timeframe of blockade, it would be impossible to uncouple the changes to host responses induced by the reduction in IFNγ signaling from changes driven by higher bacterial loads. We needed to select a blockade duration long enough to allow sufficient time for the host responses to change following reductions in IFNγR signaling but short enough that the bacteria did not have time to increase in numbers. Secondly, we reasoned that as blockade continues, a cascade of effects in the cytokine network would result in many downstream changes secondary to the loss in IFNγR signaling, and a shorter blockade would likely reveal the processes more proximal to IFNγR signaling. Four days of treatment was chosen based on the assumption that it was long enough to impact IFNγ-driven responses but quick enough that the bacteria would not have time to grow and would minimize secondary changes downstream of IFNγR blockade.

**Fig. 1:**
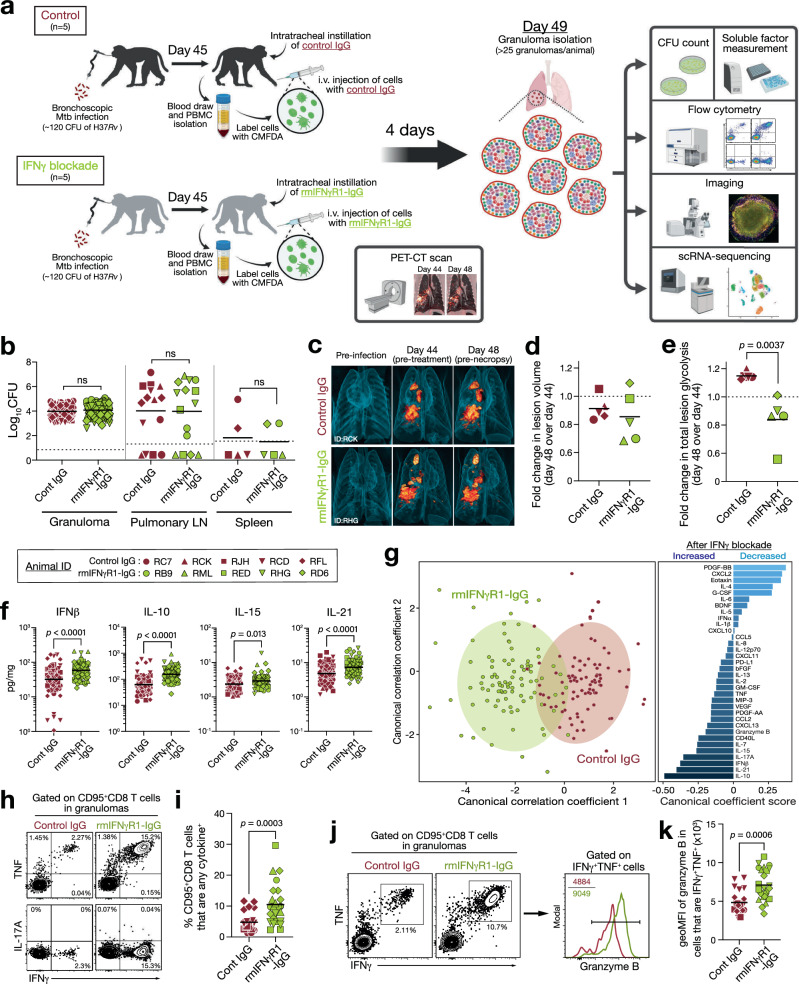
IFNγ blockade after TB granuloma formation enhances type I IFN and CD8 T cell responses in macaques. **a** Ten rhesus macaques were intrabronchially infected with 140–200 colony-forming units (CFU) of Mtb-H37*Rv* for 45 days and treated with isotype control IgG or rhesus macaque IFNγR1-IgG fusion protein (rmIFNγR1-IgG) (*n* = 5 per group). Animals were necropsied on day 49 pi and isolated tissues were subjected to downstream analysis. **b** Bacterial loads in tissues. Each symbol represents an individual tissue from each animal (granuloma, *n* = 18 per animal; pulmonary LN, *n* = 3 per animal). Dotted line indicates limit of detection. **c** ^18^FDG-PET/CT scans were performed at baseline, immediately before drug treatment (day 44) and before necropsy (day 48). Fold change over 4 days in lung lesion volume (**d**) and ^18^FDG uptake (**e**) was determined by comparing each value on day 48 over day 44 (Supplemental Data Fig. 1a, b). *n* = 5 animals per group **f**, Cytokine levels in granuloma homogenates (*n* = 18 per animal). **g** Discrimination of groups using combination of soluble factors per granuloma (*left*). sCCA was used to test whether experimental groups could be distinguished based on the concentration of all markers measured. Canonical coefficient scores were calculated to identify the soluble mediators responsible for the difference between groups in the sCCA model (*right*). Example FACS plots of cytokine staining on CD95^+^ CD8 T cells after stimulation with Mtb peptide pools (**h**) and the frequency of Ag-specific CD8 T cells that are either IFNγ^+^, TNF^+^ or IL-17A^+^ (**i**) in granulomas *n* = 5 granulomas per animal and 5 animals per group. Example FACS plots of granzyme B staining on Mtb-specific CD8 T cells (**j**) and the geometric mean fluorescent intensity (geoMFI) of granzyme B on Mtb-specific CD8 T cells (**k**) in granulomas *n* = 5 granulomas per animal and 5 animals per group. Statistical significance was calculated by two-sided Student’s *t* test. Source data are provided as a Source Data file. Created in BioRender. Barber, D. (2026) https://BioRender.com/wb056od. (2025).

Rhesus macaques were infected with Mtb and kept for 6 weeks to allow granuloma formation. On day 45 post-infection (pi), animals received either control IgG or rhesus macaque IFNγR1-IgG fusion protein (rmIFNγR1-IgG)^29^ and tissues were resected at necropsy on day 49 pi (Fig. 1a). As expected, there was no change in Mtb loads after the 4-day treatment (Fig. 1b). Lung inflammation was quantified using ^18^F-fluorodeoxyglucose (FDG)-positron emission tomography/computed tomography (PET/CT) scanning before and after the treatment (Fig. 1a, c). All animals in both groups had similar changes in the lesion volume during the treatment (Fig. 1d and Supplemental Data Fig. 1a). The total FDG uptake of control macaques increased ~15% in the 4 day treatment window, but the animals that received rmIFNγR1-IgG had a significant decrease in the lung FDG uptake (Fig. 1e and Supplemental Data Fig. 1b). Thus, this brief IFNγ blockade resulted in a rapid decrease in lung inflammation without impact on the size of lesions or bacterial growth. This allows us to study the impact of IFNγR blockade in host responses without the caveat of increased bacterial loads.

We next investigated the effect of IFNγ blockade on the production of soluble factors in granulomas. Granulomas from rmIFNγR1-IgG–treated macaques contained higher levels of IFNß, IL-10, IL-15, IL-21, CD40L and IL-17A (Fig. 1f and Supplemental Data Fig. 1c), whereas the levels of eotaxin (CCL11), CXCL2, G-CSF and PDGF-BB was significantly decreased compared with control granulomas (Supplemental Data Fig. 1c). Sparse canonical correlation analysis (sCCA) revealed that IL-21, IL-10 and IFNß were the major drivers of differences in the quality of inflammation in granulomas altered after IFNγ blockade (Fig. 1g). Next we performed flow cytometric analysis of Ag-specific T cell response to Mtb in granulomas. There was no change in the frequency of Mtb-specific CD4 T cells in granulomas (Supplementary Data Fig. 1d-f). By contrast, the frequency of Mtb-specific CD8 T cells and their expression of granzyme B were significantly increased in granulomas from IFNγ-blocked macaques compared to IgG controls (Fig. 1h-k and Supplemental Data. Figure 1g). To determine the effect of IFNγ blockade on T cell trafficking into granulomas, peripheral blood mononuclear cells (PBMCs) were labeled with a tracer dye (CMFDA) and autologously transferred back into each animal right before the infusion of drugs on day 45 pi (Fig. 1a). The migration of transferred PBMCs into granulomas was confirmed by microscopy after four days (Supplemental Data Fig. 1h). Flow cytometric quantification of the transferred cells showed no effect of IFNγ blockade on the migration of CD95^+^ CD4 and CD8 T cells into tissues (Supplemental Data Fig. 1i–l). Together these data demonstrate that brief interruption in IFNγ signaling after granuloma formation leads to increased type I IFN (IFN-I) and Mtb-specific CD8 T cell responses.

### Granuloma composition after short-term IFNγ blockade

**Fig. 2:**
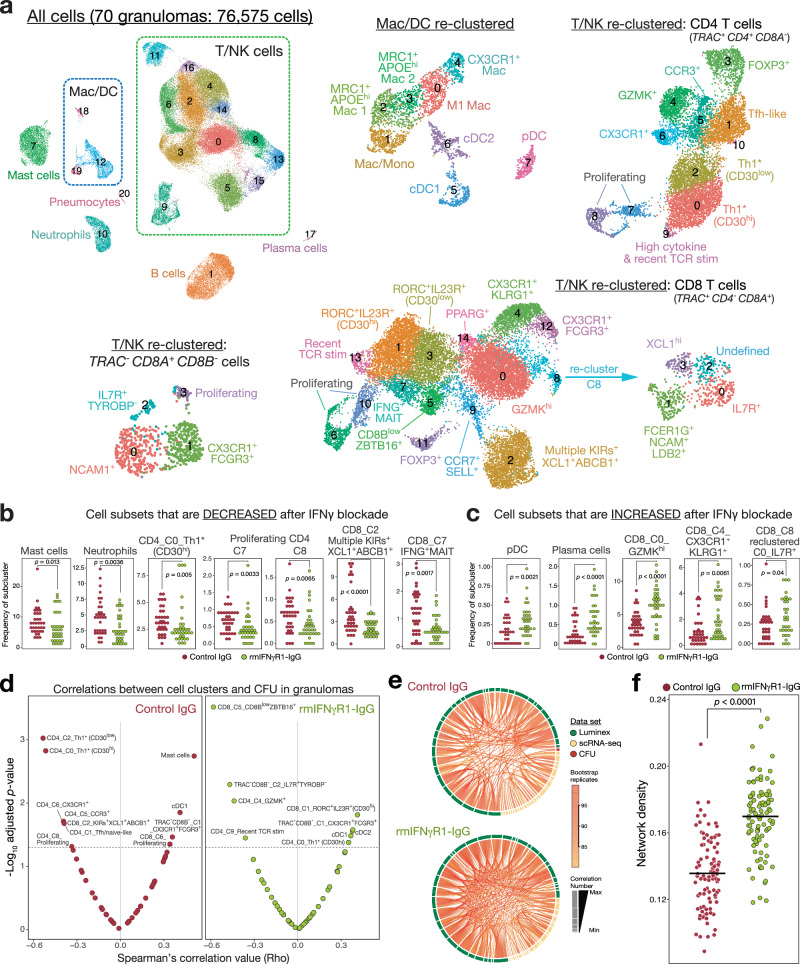
IFNγ blockade leads to increased antiviral-like responses and type-2 immunity networks in granulomas. **a** scRNA-seq analysis of 76,575 high-quality cells from macaque TB granulomas. UMAP plots show 21 major cell types and re-clustered Mac/DC, T and non-T cell subclusters. Each dot represents a single cell colored by cell identity as annotated. Cell types that are decreased (**b**) and increased (**c**) in granulomas after IFNγ blockade. *n* = 35 granulomas per group representing 7 granulomas per animal and 5 animals per group. Statistical significance was calculated by two-sided Wilcoxon test. **d** Correlations between the frequency of cell types and Mtb loads in granulomas. The dotted line indicates adjusted *p*-values < 0.05. **e** Circos plots displaying the correlations between variables of each data set. The node color corresponds to the data set of each variable, the node size represents the number of correlations of each variable, and the line color is proportional to the strength of correlations per variable. **f** The overall distribution of network density degree in 100 bootstrap replicates. Statistical significance was calculated by two-sided Wilcoxon test. Source data are provided as a Source Data file.

We next performed single-cell RNA sequencing (scRNA-seq) of the FACS-sorted live cells from 70 granulomas (total 76,575 cells, 7 granulomas per animal, 5 animals per group) (Fig. 2a). We identified 21 major cell types across all granulomas. Macs/dendritic cells (DCs), CD4 T cells, CD8 T cells and NK/ILCs were further subclustered into 8, 11, 18, and 4 distinct cell types, respectively (Fig. 2a and Supplementary Data Fig. 2a-d). IFNγ blockade resulted in a significant decrease in the proportions of mast cells, neutrophils and several T cell clusters, including CD30^hi^ (encoded by *TNFRSF8*) Th1^*^ cells (CD4_C0: *IFNG, RORA, RORC, IL23R, CCR6, CXCR3, TNFRSF8*^hi^), proliferating CD4 T cells (CD4_C7 and C8), *XCL1*^hi^ CD8 T cells (CD8_C2: *NCAM1, IKZF2, ABCB1, XCL1*^hi^), and *IFNG*^+^ MAIT cells (CD8_C7: *IFNG, ZBTB16, IKZF2, RORA, IL23R, CCR6*) (Fig. 2b and Supplemental Data Fig. 2a-d). Consistent with the cytokine/chemokine multiplex and FACS analyses, plasmacytoid DCs (pDCs), plasma cells and 3 subclusters of cytotoxic CD8 T cells (CD8_C0: *GMZK*^hi^, CD8_C4: *CX3CR1*^+^, *KLRG1*^+^ and CD8_C8-0: *IL7R*^+^) were significantly increased in IFNγ-blocked granulomas compared to IgG controls (Fig. 2c and Supplemental Data Fig. 2a-d). Quantification of the correlation between individual cell clusters and CFU in all granulomas revealed that Th1^*^ cell subclusters (CD4_C0 and C2) showed the strongest negative correlation with bacterial loads, while mast cells were strongly associated with higher CFU in the controls (Fig. 2d), which have been previously implicated in the control of Mtb infection^30–37^. IFNγ blockade drastically altered these relationships between cell clusters and bacterial load (Fig. 2d), reflecting the changes in the cellular composition in the lesions (Supplementary Data Fig. 2e).

We also performed a network analysis to examine the interconnectivity of all variables from the CFU, cytokine/chemokine multiplex and scRNA-seq analyses (Fig. 2e). The overall network density was significantly higher in rmIFNγR1-IgG-treated granulomas, indicating the treatment perturbed the system within the microenvironment (Fig. 2f). At individual variable level, we found increased connectivity of IFN-I, IL-10, cytotoxic CD8 T cell and B cell responses, whereas the network connectivity of Th1/Th1^*^ cells and M1 Macs was significantly decreased after IFNγ blockade (Supplementary Data Fig. 2f). Although we did not detect increases in the absolute levels of type-2 immune responses, the connectivity of mast cells, *MRC1/APOE*^hi^ Macs, IL-4, IL-5 and IL-13 were significantly higher in IFNγ-blocked granulomas (Supplementary Data Fig. 2f). Collectively, the data indicate that perturbation of IFNγ signaling after granuloma formation relatively quickly shifts the class of immunity from anti-microbial (helper T, granulocytes and innate-like cells) towards antiviral-like (IFN-I, killer T, pDCs and plasma cells) responses.

### Sources and targets of IFNγ in TB granulomas

**Fig. 3:**
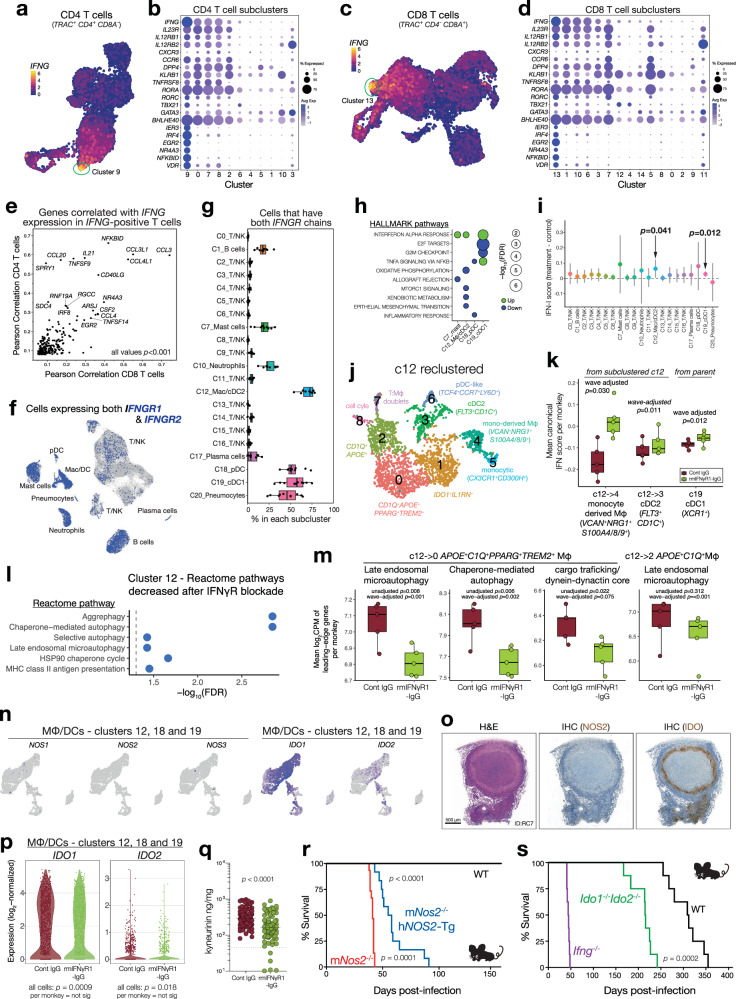
Th1*/Tc1* cell-derived IFNγ does not drive iNOS expression in macaque TB granulomas. Feature plot of *IFNG* expression by the CD4 T cell subclusters from Fig. 2a (**a**) and dot plots showing the expression of selected genes by *IFNG*^+^ CD4 T cell subclusters (**b**). Feature plot of *IFNG* expression by the CD8 T cell subclusters from Fig. 1a (**c**) and dot plots showing the expression of selected genes by *IFNG*^+^ CD8 T cell subclusters (**d**). **e** Genes expressed by *IFNG*^+^ T cells in correlation with *IFNG* expression level. **f** Feature plot of cell clusters that are both *IFNGR1*^+^ and *IFNGR2*^+^ in granulomas. **g** Frequency of *IFNGR*^+^ cells in each granuloma cell subcluster from Fig. 2a. Each dot represents a monkey with all ten animals shown. Box extends from 25th to 75th percentile and center line represents the mean. Whiskers extend to the minimum and maximum of non-outlier data points defined by values lying within Q1 − 1.5×IQR and Q3 + 1.5×IQR (where IQR = Q3 − Q1). **h** HALLMARK pathway analysis of pseudobulk differentially expressed genes in clusters 7, 12, 18 and 19, which most highly expressed both chains of the IFNγR. i. A type I IFN-induced gene expression module score was compared between cells of each cluster from treated and untreated animals. Genes included in this model are listed in the source file. Points indicate the wave-adjusted estimated difference in mean canonical type I IFN score between treatment and control for each parent cluster. Error bars denote 95% confidence intervals from monkey-level linear models (mean score ~ treatment + experimental wave). *P* values are two-sided nominal *p* values for the treatment coefficient. Error bars represent the 95% confidence intervals. **j** Cluster 12 from the parental UMAP containing macrophages and dendritic cells was reclustered. **k** Monkey-level mean type I IFN score is shown for reclustered c12 → 4 monocyte-derived macrophages, reclustered c12 → 3 cDC2, and parent c19 cDC1. Each point represents one monkey; boxes show median and interquartile range, with whiskers extending to 1.5× IQR. Wave-adjusted two-sided nominal *p* values were calculated using monkey-level linear models. **l** Reactome pathways found to be decreased in parent cluster 12. **m** Boxplots show monkey-level mean logCPM of leading-edge genes for selected pathways in c12 → 0 *APOE*^*+*^*C1Q*^*+*^*PPARG*^*+*^*TREM2*^*+*^ macrophages and c12 → 2 *APOE*^*+*^*C1Q*^*+*^ macrophages. Points represent individual monkeys; boxes show median and IQR, with whiskers extending to 1.5 × IQR. *P* values are from two-sided monkey-level linear models, shown with and without adjustment for experimental wave. **n** Expression of *NOS* isoform genes (**l**) and *IDO* isoform genes (**m**) in the Mac/DC subclusters. **o** Representative H&E staining and IHC of granulomas for iNOS (*middle*) and IDO1 (*right*). **p,** *IDO1* and *IDO2* expression in the Mac/DC cluster. Data are from all 5 monkeys/group. **q** Kyneurinin levels in granuloma homogenates. (*n* = 90 granulomas/group, *n* = 18 granulomas × 5 animals per group). **r** Survival of wild-type (WT), m*Nos2*^−/−^ or humanized iNOS mice (m*Nos2*^−/−^ / h*NOS2*-Tg) mice after Mtb infection. (*n* = 12 mice /group), *n *= 4 ×3 experiments using 4 males and 8 females. **s** Survival of WT, *Ifng*^−/−^ and *Ido1*^−/−^*Ido2*^−/−^ mice after Mtb infection. *n* = 4 ×2 experiments using 4 males and 4 females. Mice were challenged via aerosol with ~120 CFU of Mtb-H37*Rv*. Data are pooled from three independent survival experiments that gave similar results each. All scRNAseq data are from *n* = 35 granulomas per group, representing seven granulomas per animal and five animals per group. Statistical significance was calculated by two-sided Mann-Whitney *U* test. Source data are provided as a Source Data file.

We next sought to determine the sources and target cells of IFNγ within TB granulomas. *IFNG* was expressed by multiple CD4 and CD8 T cell subclusters (Fig. 3a-d and Supplementary Data Fig. 3a). The majority of *IFNG*^+^ cells were characterized by the expression of Th1^*^ cell markers, including *IL23R*, *CCR6*, *RORA* and *BHLHE40*^30–32,36^ (Fig. 3b,d). Importantly, the cells with the highest expression of *IFNG* in both CD4 and CD8 T cells (CD4_C9 and CD8_C13) were characterized by the expression of *IER3*, *IRF4*, *EGR2*, *NR4A3*, *NFKBID*, and *VDR*^38,39^ (Fig. 3b,d and Supplementary Data Fig. 3a). We have recently shown that this represents a gene signature of recent TCR activation within Mtb granulomas^40^, suggesting these small subsets are likely peptide-specific CD4 and CD8 T cells actively responding to Mtb Ags in granulomas at the time they were isolated. Furthermore, among *IFNG*^+^ T cells, *IFNG* expression levels correlated with *CCL3/3L1, CCL4/4L1, IL21, CCL20*, *TNFSF9* and *TNFSF14* expression. These data show that, as expected, *IFNG* is primarily derived from populations of TCR-stimulated Th1^*^/Tc1^*^ cells that co-express several potentially important effector molecules (Fig. 3e). In TB granulomas IFNγ-producing cells are found within the lymphocytic cuff^37,41^, and we could visualize IFNγ+ cells adjacent to CD68^+^ myeloid cells (Supplementary Data Fig. 3b). To explore cells receiving IFNγ stimulation in the microenvironment, we looked for cell types that express both *IFNGR1* and *IFNGR2* as a functional receptor (Fig. 3f). Approximately 75% of Mac/DC clusters were *IFNGR1/2*^+^, whereas ~20% of the B cell, neutrophil and mast cell clusters expressed both receptor genes (Fig. 3g). Notably, while rare in our dataset, pneumocytes also showed a high expression of IFNγR (Fig. 3g).

### Differential gene expression in granulomas after IFNγ blockade

We next performed differential expression analysis comparing monkey-level pseudobulk profiles from control and rmIFNγR1-IgG-treated granulomas. We first focused on the identification of Hallmark pathways that changed in the clusters that expressed high levels of IFNγR. The most apparent effect of blockade was an increase in type I IFN signaling in cluster 7 (mast cells), cluster 12 (Macs/cDC2) and cDC1 cells (Fig. 3h). We then created a type I IFN-driven gene module score and measured the difference between treated and control granuloma cells. We found a nominal increase in type I IFN-induced gene signatures in cluster 12 (Macs/cDC2s) and cDC1 cells (Fig. 3h). Neutrophils, mast cells and pDCs trended toward increased type I IFN-driven gene signatures, but these changes did not reach statistical significance. Since cluster 12 was a heterogeneous population, we next subclustered c12 and identified several subsets of macrophages and dendritic cells (Fig. 3j). We applied the same type I IFN gene signature to these subclusters, and found that, in addition to cDC1 cells from the parental UMAP, cDC2 cells and a population of monocyte-derived-like macrophages (*VCAN*^*+*^*NRG1*^*+*^*S100A4/8/9*^*+*^) from the subclustered c12 population displayed enhanced type I IFN-driven gene signatures (Fig. 3k). These findings are consistent with our detection of increased IFNβ multiplex ELISA as well as previous results finding an IFN-driven gene signature associated with detrimental outcomes of Mtb infection^42–45^.

We next focused on pathways that were decreased after IFNγR blockade. cDC1 cells showed a significant reduction in Hallmark pathways related to cell cycle after IFNγR blockade (Fig. 3h). Cluster 12 (Macs/cDC2s), showed the greatest number of Hallmark pathway differences, so we next focused on these cells. Pseudobulk differential expression analysis of control and treated cluster 12 cells identified several reduced Reactome pathways related to mitophagy, endosomes, and MHC class II antigen presentation after IFNγR blockade (Fig. 3l). To ask whether these pathways were differentially affected across cluster 12 subsets, we extracted the leading-edge genes underlying those pathways and calculated their pseudobulk mean expression for each subcluster. Cluster c12->0 (*APOE*^*+*^*C1Q*^*+*^*PPARG*^*+*^*TREM2*^*+*^) cells showed a nominal reduction in Reactome pathways associated with autophagy and cargo trafficking. We also found a reduction in the leading-edge genes of the late endosomal microautophagy pathway in c12->2 cells, which also expressed *C1Q* and *APOE*, but had fewer distinguishable DEGS (Fig. 3m). These results are consistent with previous reports that IFNγ can drive autophagy in Mtb infected cells^46–48^. Collectively, these data show that IFNγR blockade results in increased type I IFN-driven gene expression in dendritic cells and *VCAN*^*+*^ macrophages, and decreases in genes associated with autophagy and intracellular cargo trafficking in *C1Q*^+^*APOE*^+^ macrophages.

We next directly examined expression of nitric oxide synthase, given its primary role in IFNγ-mediated control of Mtb infection in mice. Strikingly, we observed little-to-no expression of *NOS2, NOS1* and *NOS3* in myeloid cells in macaque granulomas (Fig. 3n). However, the IFN-inducible, tryptophan catabolizing enzyme *IDO1* was highly expressed across multiple Mac/DC subclusters(Fig. 3n). *IDO2* was also found, but its expression was more selective (Fig. 3n). Immunohistochemistry (IHC) analysis confirmed the lack of iNOS and high levels of IDO-1 in granulomas (Fig. 3o). Expression of *IDO1* and *IDO2* (Fig. 3p) and the levels of their product kynurenine (Fig. 3q) were only modestly decreased in granulomas after 4 days of IFNγ blockade. We next examined the role of iNOS and IDO in Mtb infection using mouse models. As has been extensively documented, *Nos2*-deficient (m*Nos2*^*−/−*^) mice were extremely susceptible to Mtb infection (Fig. 3r). To ask if the regulation of NO induction may contribute to the differences between species, we next examined Mtb infection in mice in which the murine *Nos2* locus has been removed and the human *NOS2* gene and promoter has been inserted (m*Nos2*^−/−^h*NOS2*-Tg)^17^. We found that while humanized iNOS mice displayed improved survival compared to mice completely deficient in *Nos2*, they succumbed much earlier than mice with a wild-type murine *Nos2* gene and promoter (mean survival time: 293 days [wild-type] vs 41 days [*mNos2*^−/−^] vs 56 days [m*Nos2*^−/−^/h*NOS2*-Tg]) (Fig. 3r). In contrast, mice lacking both IDO1 and IDO2 (*Ido1*^−/−^*Ido2*^−/−^) were more susceptible to Mtb infection compared to controls (mean survival time: 310 days [wild-type] vs 215 days [*Ido1*^−/−^*Ido2*^−/−^]) (Fig. 3s). These data may suggest that high resistance of C57Bl/6 mice to Mtb infection compared to humans and non-human primates (NHPs) is in part due to the IFNγ responsiveness of the *Nos2* promoter and the production of high levels of NO at sites of Mtb replication, illustrating a fundamental difference in IFNγ-mediated host resistance against TB between mice and primates. IDO1/2, on the other hand, is abundant in NHP granulomas and has a relatively minor role in protection in mice compared to iNOS.

### IFNγ blockade from the onset of Mtb infection

**Fig. 4:**
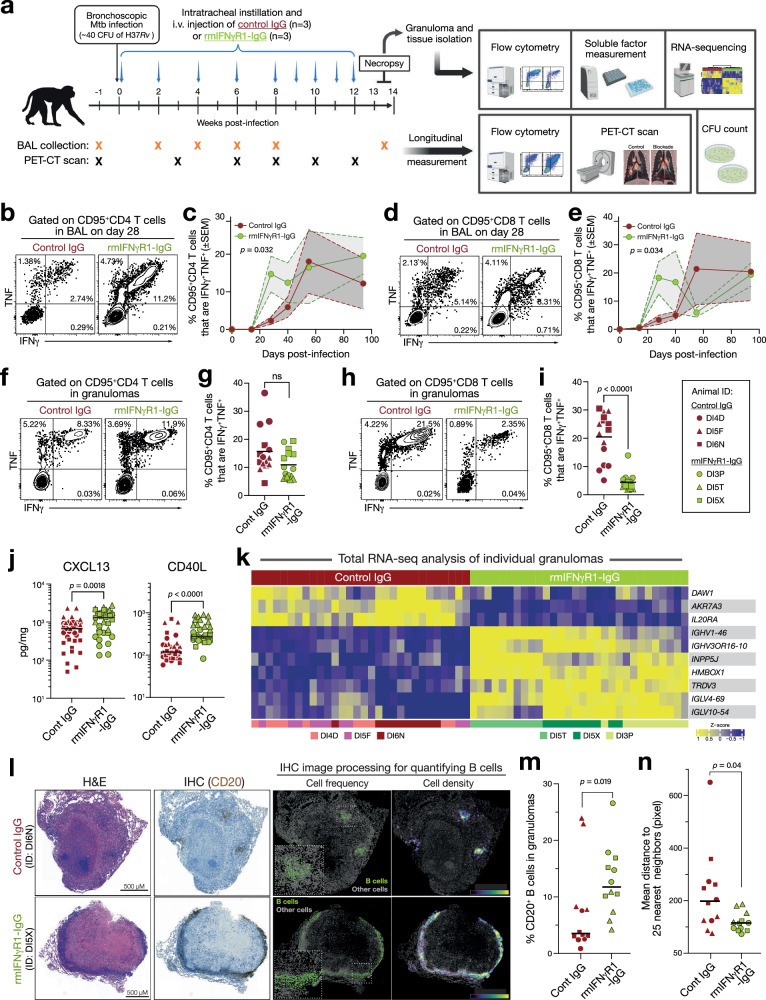
IFNγ blockade from the beginning of infection alters the kinetics of Mtb-specific T cell responses and B cell distribution in TB granulomas. **a** Six rhesus macaques were infected with 30–60 CFU of Mtb-H37*Rv* and treatment with isotype control IgG or rmIFNγR1-IgG (*n* = 3 per group) was started on the day of infection. Animals were necropsied at 13–14 weeks p.i. Example FACS plots of cytokine staining on CD95^+^ CD4 T cells after stimulation with Mtb peptide pools (**b**) and kinetics of the frequency of Ag-specific CD4 T cells that are IFNγ^+^ and TNF^+^ (**c**) in BAL. Error bars represent the range. Statistical significance was calculated by Two-way ANOVA. Example FACS plots of cytokine staining on CD95^+^ CD8 T cells after stimulation with Mtb peptide pools (**d**) and kinetics of the frequency of Ag-specific CD8 T cells that are IFNγ^+^TNF^+^ (**e**) in BAL. Error bars represent the range. 05 Statistical significance was calculated by Two-way ANOVA. Example FACS plots of cytokine staining on CD95^+^ CD4 T cells after stimulation with Mtb peptides (**f**) and the frequency of IFNγ^+^TNF^+^ CD4 T cells (**g**) in granulomas. *n* = 15 granuloma per group. Example FACS plots of cytokine staining on CD95^+^ CD8 T cells after stimulation with Mtb peptides (**h**) and the frequency of IFNγ^+^TNF^+^ CD8 T cells (**i**) in granulomas. *n* = 15 granuloma per group. ns, non-significant, **** *p* < 0.0001 by Student’s *t* test. **j** Levels of CXCL3 and soluble CD40L in granuloma homogenates (*n* = 12 per animal). ** *p* < 0.01, **** *p* < 0.0001 by Student’s *t* test. **k** Bulk RNAseq analysis of individual granulomas (*n* = 10 per animal). Heatmap showing a total of 10 DEGs with |log2 fold-change|>1 and false discovery fate (FDR) < 0.05. **l** Representative images of granulomas and image processing for quantification of B cells. Scale bars represent 500 μM. The frequency (**m**) and spatial distribution (**n**) of B cells in individual granulomas. *n* = 12 granulomas per group. Statistical significance was calculated by two-sided Mann-Whitney One-tailed U test, with the alternative hypotheses defined a priori (**m**: IFNγR1-IgG > isotype; **n** isotype > IFNγR1-IgG). Source data are provided as a Source Data file.

To examine the role of IFNγ over the entire course of infection, we next treated macaques with control IgG or rmIFNγR1-IgG from day 0 through ~13-14 weeks pi (Fig. 4a). We first profiled the kinetics of T cell responses to Mtb in bronchoalveolar lavages (BAL). At ~4 weeks pi, there was a significant increase in the frequency of both Mtb-specific CD4 and CD8 T cells in rmIFNγR1-IgG–treated BAL compared to IgG controls (Fig. 4b-e). At necropsy, Mtb-specific CD4 T cell response per granuloma was similar between control IgG and IFNγ-blocked groups (Fig. 4f,g), while the frequency of Mtb-specific CD8 T cells was markedly decreased in IFNγ-blocked granulomas (Fig. 4h,i).

We next performed a cytokine/chemokine multiplex analysis of granuloma homogenates. There was an overall decrease in levels of multiple soluble factors measured, in particular the IFNγ-inducible chemokine CXCL11 (Supplemental Data Fig. 4a). Only two analytes, CXCL13 and soluble CD40L, were increased in rmIFNγR1-IgG–treated granulomas compared to controls (Fig. 4j). Bulk RNAseq analysis of individual granulomas identified three genes whose expression was largely ablated by IFNγ blockade: *IL20RA, DAW1* and *AKR7A3* (Fig. 4k). Seven genes were found to be highly upregulated in granulomas after IFNγ blockade including four immunoglobulin genes (Fig. 4k). Given the increase in the B cell-recruiting chemokine CXCL13 and elevated expression of BCR genes, we next performed IHC staining for CD20 and quantified the frequency and spatial distribution of B cells in granulomas (Supplemental Data Fig. 4b, c). B cells accounted for ~4% of all cells in control IgG granulomas and resided predominantly in follicular-like structures in the lymphocyte cuff (Fig. 4l-n). Intriguingly, IFNγ-blocked granulomas exhibited an increased presence of B cells (~12% of all cells) and were distributed in densely packed rings circumscribing the periphery of granuloma (Fig. 4l-n). Despite the increased presence of B cells, Mtb-specific IgG titers were similar between 2 groups of granulomas (Supplemental Data Fig. 4d). Collectively, IFNγ blockade started at the time of Mtb infection altered the kinetics of Mtb-specific T cell responses and limited the accumulation of B cells in granulomas. Interestingly, B cell depletion in vaccinated macaques limits the recruitment of CXCR5^+^ CD4 T cells, formation of granuloma-associated lymphoid tissue and bacterial control^49^.

### Impact of IFNγ blockade on TB disease and bacterial growth in macaques

**Fig. 5:**
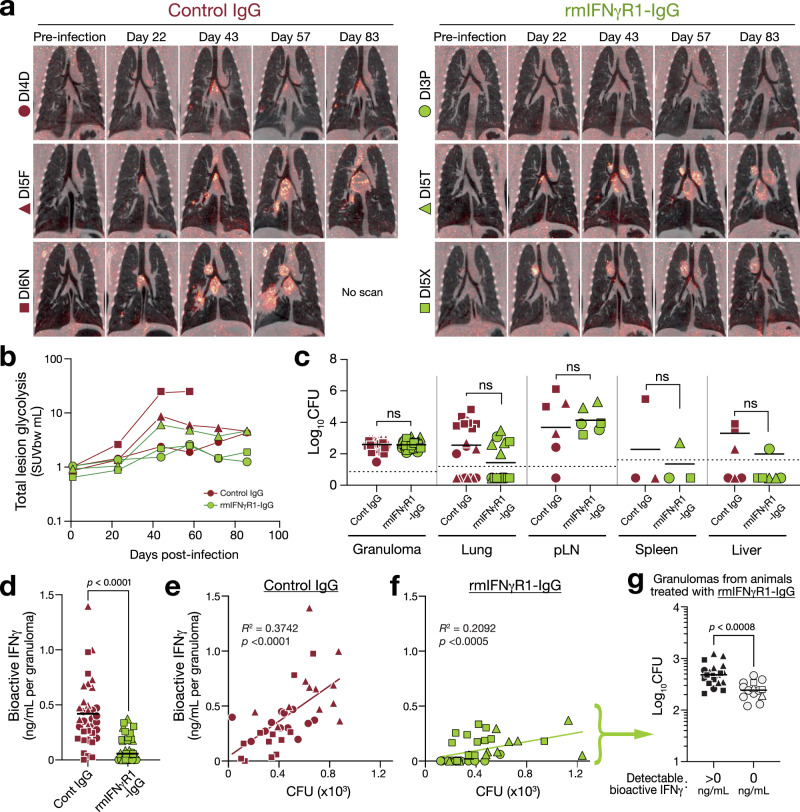
IFNγ blockade from the beginning of infection did not affect lung disease and Mtb loads in macaques. **a** PET/CT images showing lung FDG signal from pre-infection and 12 weeks pi. **b** Lung inflammation (total ^18^FDG activity) over the course of infection as measured by serial PET/CT scanning. **c**, Bacterial loads in tissues. Each symbol represents an individual tissue from each animal with 5 animals per group (granuloma, *n* = 12 per animal, *n* = 36 per group; lung piece, *n* > 5 per animal, *n* = 20 pieces from controls and 17 from treated; pulmonary LN, *n* = 2 per animal and *n *= 6 per group; liver piece, *n* = 2 per animal and *n* = 6 per group). Dotted line indicates limit of detection. **d** Levels of bioactive IFNγ in granuloma homogenates (*n* = 12 per animal). Correlations between the bioactive IFNγ levels and Mtb loads in granulomas homogenates from animals treated with control IgG (**e**) and rmIFNγR1-IgG (**f**). **g** Comparison of bacterial loads in granulomas with/without bioactive IFNγ activity from rmIFNγR1-IgG–treated macaques in Fig. 5f. *n* = 35 granulomas. Statistical significance was calculated using two-sided by Student’s *t* test. Source data are provided as a Source Data file.

To assess the disease outcome of Mtb infection after long-term IFNγ blockade, we monitored lung inflammation of infected macaques by serial ^18^FDG-PET/CT imaging (Fig. 5a). Consistent with previous findings in rhesus macaques^50,51^, lung inflammation associated with increased ^18^FDG uptake was evident by ~6 weeks p.i. and persisted until necropsy in IgG control animals (Fig. 5b). Unexpectedly, rmIFNγR1-IgG–treated animals exhibited similar levels of lung ^18^FDG uptake compared to IgG controls for the duration of the experiment (Fig. 5b). Surprisingly, we observed no difference in Mtb burdens between two groups in individual granulomas, lung, lymph node, spleen and liver tissues (Fig. 5c).

To validate the efficacy of IFNγ blockade, we quantified the concentration of biologically functional IFNγ in granuloma homogenates by IFNγ reporter cell assay^29^. In IgG control granulomas the median bioactive IFNγ level was 421 pg/mL per granuloma (Fig. 5d). By contrast, the level of bioactive IFNγ was 86 pg/mL per granuloma in IFNγ-blocked granulomas. Importantly, nearly half of granulomas in treated animals had no detectable bioactive IFNγ while functional IFNγ was detectable in all but one control granuloma (14 out of 36 granulomas [rmIFNγR1-IgG] vs 1 out of 36 granulomas [control IgG]) (Fig. 5d). Thus, the treatment effectively inhibited biological activity of IFNγ within granulomas, but it did not achieve complete neutralization in vivo. To address whether the extent of blockade was sufficient to observe differences in bacterial loads, we next asked whether the levels of bioactive IFNγ correlated with Mtb loads in individual granulomas. In IgG control granulomas, we observed a positive correlation between IFNγ concentration and CFU (Fig. 5e), consistent with previous findings of similar trends between the magnitude of CD4 T cell response and Mtb burden^22,50,52^. IFNγ-blocked granulomas also showed a positive correlation between bioactive IFNγ and CFU (Fig. 5f). Among granulomas from treated animals, the granulomas with undetectable IFNγ bioactivity had significantly lower bacillary loads compared to granulomas that contained residual bioactive IFNγ (Fig. 5g). Thus, there was no indication that reducing the levels of IFNγ down to undetectable levels resulted in increased bacterial loads. Instead, the lower the levels of IFNγ present in the granuloma, the lower bacterial loads we found. Together, these data demonstrate that IFNγ can be suppressed down to very low levels without exacerbating Mtb burden or disease in rhesus macaques.

### Mtb-specific T cells in NTM patients with anti-IFNγ autoAbs

**Fig. 6:**
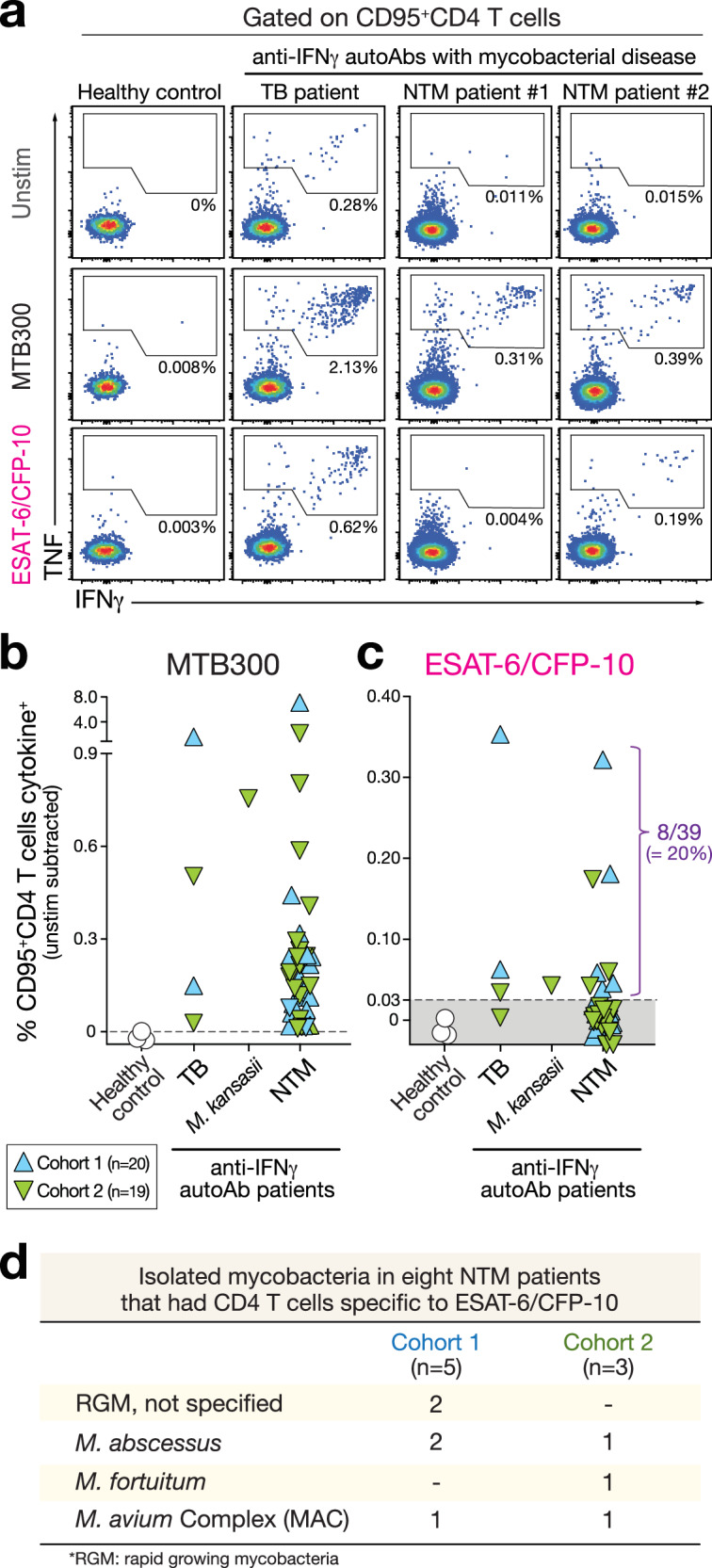
Mtb-specific T cell responses without clinical TB in NTM patients with anti-IFNγ autoAbs. **a** Example FACS plots of cytokine staining on CD95^+^ CD4 T cells after stimulation of PBMCs with mycobacterial Ag peptide pools. Healthy control, *n* = 3; anti-IFNγ autoAbs patients with TB, *n* = 4; anti-IFNγ autoAbs patients with *M. kansasii*, *n* = 1; anti-IFNγ autoAbs patients with NTM, *n* = 39 (cohort 1, *n* = 20; cohort 2, *n* = 19). **b** Frequency of CD4 T cells that are IFNγ^+^ or TNF^+^ after stimulation with MTB300 peptide pools. **c** Frequency of CD4 T cells that are IFNγ^+^ or TNF^+^ after stimulation with ESAT-6/CFP-10 peptide pools. Dashed line indicates an arbitrary threshold for quantification of ESAT-6/CFP-10–specific CD4 T cells (Supplementary Data Fig. 5). **d** Mycobacterial strains isolated from the anti-IFNγ autoAbs patients that had CD4 T cell response to ESAT-6/CFP-10. Source data are provided as a Source Data file.

Individuals who develop neutralizing autoAbs against IFNγ are much more likely to develop extrapulmonary NTM infections as compared to pulmonary TB^24,25^. This may indicate that IFNγ is not always required for control of pulmonary Mtb infection, or it may reflect a major difference in levels of exposure to the pathogens. To distinguish between these possibilities, we sought to determine if NTM patients with neutralizing anti-IFNγ autoAbs who developed NTM disease rather than TB had been exposed to Mtb. PBMCs from Southeast Asian NTM patients with anti-IFNγ autoAbs were analyzed for Mtb-specific T cell responses (Cohort 1: 20 patients living in Thailand and Taiwan^24^, Cohort 2: 19 patients living in the United States). Cells were stimulated with either the MTB300 peptide megapool, comprising peptides expressed by many mycobacteria, or with peptide pools from ESAT-6 and CFP-10, which are only expressed in *M. tuberculosis* and a few NTM, including *M. kansasii*^53^. Ag-specific CD4 T cells were measured by intracellular staining for IFNγ and TNF. Following stimulation with the MTB300 megapool, we observed mycobacteria-specific T cell responses in all NTM patients and control TB patients but not healthy controls (Fig. 6a, b). As expected, ESAT-6/CFP-10–specific T cells were detected in TB patients and a patient infected with *M. kansasii* (Fig. 6c). Notably, eight out of 39 patients with NTM disease also showed ESAT-6/CFP-10–specific T cell responses at levels similar to patients with Mtb or *M. kansasii* infection (Fig. 6c). Microbiological analysis revealed the primary causative agents in those patients were neither Mtb or ESAT-6/CFP-10–expressing slow-growing NTM^54,55^ (Fig. 6d). Thus, some individuals with neutralizing anti-IFNγ autoAbs develop NTM disease but not TB, despite the immunological evidence of exposure to Mtb.

## Discussion

IFNγ has long been considered as a central host protective cytokine in Mtb infection. This study provides insight into the role of IFNγ in the orchestration of host responses in TB granulomas as well as its relative importance in host resistance to Mtb infection.

We find that IFNγ blockade after granuloma formation results in rapid increases in; (*i*) IFNβ, IL-10, IL-15, granzyme B, and IL-21, (*ii*) pDCs, plasma cells and Mtb-specific CD8 T cells and (*iii*) IFN-I–driven gene expression in myeloid cells. Differential gene expression analysis found that IFNγ blockade resulted in increased type I IFN responsiveness primarily in dendritic cells and *VCAN*^*+*^ macrophages. Our data did not reveal how IFNγ negatively regulates IFNβ in TB granulomas. We could not detect IFN-I in our scRNA-seq analysis, so it is also not clear which cells produced IFNβ. Nonetheless, IFN-I, IL-10 and pDCs have been shown to be detrimental during Mtb infection^43,56,57^. We also observed decreases in helper and unconventional T cells and granulocytes as well as decreases in autophagy-related pathways in *C1Q*^*+*^*APOE*^*+*^ macrophages. Several classic IFNγ-driven genes that we expected to change in Mac/DC subclusters did not, at least not after only 4 days of blockade. For example, *IDO1* and *CXCL9/10/11* expression levels changed little or were not different in controls vs treated granulomas. Despite their opposing roles in protection from mycobacteria, there is significant overlap between type I and type II IFN-inducible genes, so the increase in IFNβ may have masked some of the changes that occurred when IFNγ was reduced. This may also reflect IFNγ-induced transcriptional memory^58^ and indicate that IFNγ blockade in IFNγ-primed macrophages may take longer than 4 days to observe changes in expression of some genes. These results show that IFNγ may, at least in part, regulate the class of inflammation, i.e., by promoting antimicrobial responses while limiting detrimental IFN-I and antiviral-like responses.

IFNγ is clearly required for human resistance to mycobacteria^23,59^, and we were surprised to find no impact of IFNγ blockade on disease or bacterial loads in the infected macaques. Using a sensitive reporter cell assay, we found that we had not completely neutralized bioactive IFNγ in all granulomas, so it is possible that the amount of cytokine still available during blockade, although greatly reduced, was sufficient for host protection. This observation may align with some findings in humans. Most individuals with MSMD due to IFNγ/IFNγR deficiency develop extrapulmonary NTM infections^23^. Similarly, adults with high titers of neutralizing Abs against IFNγ develop extrapulmonary NTM infection far more frequently than pulmonary TB^24,25^. Moreover, here we showed that some of these patients have been exposed to Mtb but never developed TB. The “resister” endotype shows that Mtb-specific CD4 T cells always polarize into IFNγ-secreting cells during Mtb infection, at least in some individuals^26–28^. A recent study also demonstrated that non-Th1–polarized T cell responses are associated with resistance to Mtb in certain strains of collaborative cross mice^60^. We hypothesize that while genetic ablation of IFNγ leads to extreme susceptibility to mycobacterial infection, small amounts of IFNγ (i.e., below what we could achieve with the approach used in this experiment) may be sufficient for normal host resistance to pulmonary TB in adult humans and NHPs. It is also possible that IFNγ may play a larger role in resistance to Mtb infection earlier in life. In fact, we cannot rule out the possibility that the major role for IFNγ in resistance to TB is prior to rather than during infection, perhaps by the training of macrophages during early life^61^.

In contrast to the limited role of IFNγ in control of pulmonary TB in macaques and humans, *Ifng* gene deficiency leads to extreme disease in mouse models of tuberculosis. The prominent dependence of host resistance on IFNγ in the mouse model of TB is largely attributed to the induction of iNOS by IFNγR signaling, as *Nos2*^*−/−*^ mice are also hypersusceptible to Mtb infection. In contrast to mice, we find very little evidence of *NOS1/2/3* gene expression in NHP granulomas. Moreover, we find that humanized iNOS mice are extremely susceptible to Mtb infection. We propose that the relatively high degree of resistance of mice to Mtb infection as compared to humans and NHP is primarily due to the ability of IFNγ to drive iNOS expression in mice. In other words, mice are actually very susceptible hosts if not for the unique ability of IFNγ to potently upregulate iNOS in this species.

There are several experimental caveats in our studies to consider. A neutralizing monoclonal Ab against IFNγ rather than the cytokine receptor fusion reagent used here may have more effectively reduced IFNγR signaling closer to zero. The use of another species of NHPs or Mtb strains may have shown a larger impact of IFNγ blockade on the outcome of infection. In the short-term study, we do not know the extent of IFNγ neutralization. While in the long-term blockade experiment, we were able to directly show effective cytokine neutralization in granulomas, we do not know if the animals mounted an anti-drug antibody response. It is also possible that longer blockade would have eventually led to exacerbated disease. IFNγ blockade may have a more apparent role during amnestic responses following challenge infection of vaccinated hosts. We cannot exclude the possibility that IFNγ appears to have less of a role in Mtb-infected macaques because Mtb itself or local immunoregulatory pathways inhibit IFNγ responses in the first place^37,62–64^. The unexpectedly low impact of IFNγ blockade on bacterial loads could reflect poor co-localization of IFNγ-producing cells with IFNγR-expressing infected macrophages, as most CD4 T cells are not close to the infected core of the granuloma^50^. Lastly, it is also possible that the accelerated accumulation of Mtb-specific T cells in the airways during IFNγ blockade could have countered the detrimental effects of the loss of IFNγ signaling.

The largest population of CD4 T cells was *IL23R*^*+*^*IL12R*^*+*^*CCR6*^*+*^*CD26*^*+*^*CD161*^*+*^, expressed the transcription factors *RORA, BHLHE40* and to a lesser extent *RORC and TBX21*, indicating these are Th1^*^ cells. In humans and NHPs, Mtb-specific CD4 T cells with these markers have been shown to correlate with better control of the infection^31–36,65,66^, and here we found that Th1^*^ cells in granulomas negatively correlate with bacterial loads. We have recently shown that Mtb-specific Th1^*^ cells express many different effector molecules that may contribute to suppression of bacterial growth^40^. For example, T cell-derived TNF, GM-CSF and IL-21 may contribute to IFNγ-independent control of Mtb infection in mouse models^22,67–73^. Apart from IFNγ, there are many T cell-derived molecules that have not been explored in host resistance to tuberculosis.

The long-held view that IFNγ is the primary mediator of adaptive immunity to Mtb infection has been, for the most part, based on the extreme susceptibility of IFNγ-deficient mice to Mtb infection and the observations of non-tuberculous mycobacterial infections in individuals with defects in IFNγ-mediated immunity. However, the data shown here and elsewhere find that macaques and some humans can resist pulmonary Mtb infection despite neutralization of IFNγ to very low levels. We also propose an explanation as to why mice do not recapitulate the role of IFNγ humans and macaques. In mice, but not humans or macaques, IFNγ drives enormous production of antimicrobial reactive nitrogen species through the induction of iNOS. Thus, the major mechanisms of adaptive immunity to Mtb infection in humans and macaques remain unknown. Identification of novel T cell effector molecules that contribute to control of Mtb infection may provide important mechanism-based correlates of protection to guide the development of new TB vaccines and host-directed therapies.

## Methods

### Ethics statement

All animal procedures were approved by the National Institute of Allergy and Infectious Diseases (NIAID) Division of Intramural Research Animal Care and Use Committee (ACUC) under study proposal LPD-25E and were performed in accordance with the Animal Welfare Act, the Guide for the Care and Use of Laboratory Animals, and all applicable institutional regulations, standards, and policies. Human studies were conducted under Institutional Review Board-approved protocols NCT00814827, NCT00018044, NCT00001355, and NCT01212003. Written informed consent was obtained from all participants in the human cohorts. Healthy control whole blood samples were obtained from anonymous donors through the NIH Blood Bank in accordance with institutional policies.

### Biosafety

All work with live Mtb or animals infected with Mtb was conducted in a Biosafety Level 3 (BSL3) or Animal Biosafety Level 3 (ABSL3) facility. All protocols and standard operating procedures (SOPs) were approved by the Institutional Biosafety Committee and the Department of Safety. All ABSL3 animals were housed in biocontainment isolator caging, and handling of macaques was done only on anesthetized animals. All personnel working with Mtb are enrolled in the NIH Biosurety program, receive initial protocol and SOP training and undergo annual refresher training thereafter.

### Rhesus macaques

Sixteen healthy, 3-year-old male rhesus macaques, originally from the National Institute of Allergy and Infectious Diseases (NIAID) breeding colony on Morgan Island, were selected for this study and were tuberculin skin test negative. Animals were housed in NHP biocontainment racks and maintained in accordance with the Animal Welfare Act, the Guide for the Care and Use of Laboratory Animals and all applicable regulations, standards, and policies in a fully AAALAC International-accredited ASBL3 vivarium. Euthanasia methods were consistent with the AVMA Guidelines on Euthanasia and endpoint criteria listed in the NIAID DIR ACUC-approved animal study proposal LPD-25E.

### Human subjects

In this study, two existing historical NTM cohorts were used. Participants (*n* = 20) in Cohort 1 residing in Thailand and Taiwan^24^ were consented onto an Internal Review Board (IRB)-approved protocol NCT00814827. Cohort 2 was comprised of subjects (*n* = 19) provided written informed consent onto IRB-approved protocols NCT00018044 and NCT00001355, and all but one were born outside of the U.S. (Thailand, Philippines, Vietnam, Cambodia, China) but currently residing within North America. All patients in both cohorts were of Southeast Asian descent, followed either in Thailand or at the NIH. The diagnosis of, and functional assay of anti-IFNγ autoAbs were made by a luciferase immunoprecipitation system as previously described^74^. The diagnosis of NTM was culture-proven and when possible, biochemical or genetically based identification of species was performed. The TB cohort was consented on IRB-approved protocols NCT01212003 and NCT00001355. TB diagnosis was based on culture-proven Mtb or smear-positive results for acid-fast bacilli. Healthy control whole blood was collected from anonymous donors at the NIH Blood Bank. PBMCs were isolated by Ficoll-Paque (GE Life Sciences) density centrifugation and stored at −80 °C until later use. The Thai samples were a convenience sample taken in an anonymized non-random fashion based on the most recent shipment date to the U.S. Frozen PBMCs were thawed and rested overnight in X-VIVO 15 media (Lonza) supplemented with 10% FCS at 37 °C before in vitro stimulation with peptide pools.

### Mice

Wild-type C57BL/6 mice were purchased from Taconic Farms (Germantown, NY). m*Nos2*^−/−^ and *Ifng*^−/−^ mice were obtained through a supply contract between the NIAID and Taconic Farms. Humanized NOS2 transgenic (m*Nos2*^−/−^/h*NOS2*^−/−^) mice^17^, that express a functional human *NOS2* gene and promoter, were kindly provided by Dr. Carol A. Colton (Duke University, North Carolina), and *Ido1*^−/−^*Ido2*^−/−^^75^ mice were a kind gift of Dr. Peter Murray (St Jude Children’s Research Hospital, Memphis, Tennessee). h*NOS2*-Tg were crossed to m*N*os*2*^−/−^ mice, and double transgenic mice were used in experiments. All mice were maintained in the NIAID animal facility. All mice were socially housed in individually ventilated solid bottom cages on hardwood bedding (Sani-Chips, ASAP) that has a relative negative pressure micro-environment to the surrounding room. Mice were maintained on a 12:12-h light:dark cycle, at 72 ± 3 °F ambient temperature, and 30–70% relative humidity. All mice were given nesting pads and chew sticks for environmental enrichment. All animals (9–12 weeks old, sex matched) were housed in an AAALAC International-accredited ABSL3 NIAID facility in accordance with the National Research Council Guide for the Care and Use of Laboratory Animals. All procedures were performed as listed in the NIAID DIR ACUC-approved animal study proposal LPD-24E.

### Mtb infection and IFNγ blockade

Rhesus macaques were anesthetized, and 2 mL of PBS containing the H37*Rv* strain of Mtb (~140–200 CFU for short-term or ~30–60 CFU for long-term blockade) was bronchoscopically instilled into the right lower lung lobe. Infection dose was confirmed by plating of aliquots onto 7H11 agar plates supplemented with oleic acid–albumin–dextrose–catalase (OADC) (Difco). For short-term blockade, animals were treated with rhesus macaque IFNγR1-IgG fusion protein (rmIFNγR1-IgG)^29^ or rhesus macaque IgG_4_ isotype control antibody (clone DSPR4) intravenously at 10 mg/kg of body weight (in 30 mL of PBS) and intratracheally at 1 mg/kg of body weight (in 2 ml of PBS) on day 45 pi (Fig. 1a). In long-term blockade experiment, animals received the same treatment bi-weekly for the first 8 weeks and then every week for additional 4 week before necropsy at week 13–14 pi (Fig. 4a).

For Mtb infection in mice, animals were exposed to ~120 CFU of Mtb-H37*Rv* using an inhalation exposure system (Glas-Col LLC). Bacterial loads were measured in tissue homogenates by serial dilution on 7H11-OADC agar plates.

### PET/CT scanning and data analysis

Rhesus macaques were imaged with an optimized ^18^FDG dose (0.5 mCi/kg) administered intravenously as previously described^50–52^. In brief, A 360-projection CT scan of the lungs was acquired during a ~ 50-s breath hold on a LFER 150 PT/CT scanner (Mediso Inc, Hungary). A 20-min PET dataset/per field of view was acquired during mechanical ventilation and the raw CT and PET data were reconstructed using the Nucline software (Mediso Inc.) to create individual DICOM files that were co-registered using MIM Maestro (v.6.2, MIM Software Inc). A lung volume of interest (VOI) was defined on the CT image and the VOI was transferred to the PET image as previously described to determine the total ^18^FDG uptake referred to as the lung total lesion glycolysis (TLG). The analysis of disease burden included abnormal TLG of the lung, and a consistent region of the hilar and subcarinal lymph nodes (LNs) of each animal with standardized uptake value above background. Two readers independently performed image analysis for each animal. Three-dimensional projections were generated using Osirix v.5.9 software (Pixmeo, Switzerland).

### Cell isolation and in vitro stimulation

Blood samples were collected in EDTA tubes, and PBMCs were isolated by Ficoll-Paque density centrifugation. For autologous transfer, isolated PBMCs were labeled with 0.5 mM CMFDA (Thermo Scientific) per manufacturer’s instructions and infused back into animals intravenously. BAL samples were passed through a 100-µm cell strainer, pelleted, and counted for analysis. Granulomas were individually resected from the lungs and were pushed through a 100-µm cell strainer. LNs, spleens and liver tissues were dissociated using a GentleMACS Tissue Dissociator (Miltenyi Biotec). Aliquots from all samples were serially diluted and plated on 7H11-OADC agar plates for CFU measurement. Cells were stimulated in X-VIVO 15 media supplemented with 10% FCS at 37 °C with either a mixture of CD4 (MTB300) and CD8 Mtb peptide megapools (2 µg/ml and 1 µg/ml, respectively), or a mixture of ESAT-6 and CFP-10 peptide pools (2 µg/ml and 2 µg/ml, respectively), MTB300 alone for 6 h in the presence of brefeldin A and monensin (eBioscience).

### Flow cytometry

Fluorochrome-conjugated antibodies used for FACS analysis are listed in Supplementary Table 2. Surface Ags and dead cells were stained in PBS + 1% FCS + 0.1% sodium azide for 20 min at 10 °C. For intracellular cytokine and transcription factor staining, cells were fixed and permeabilized with the Foxp3 Transcription Factor Staining Buffer Kit (eBioscience) and stained for 30 min at 10 °C. Samples were acquired on a FACSymphony A5 (BD Biosciences), and data were analyzed using FlowJo 10 (Tree Star). The gating strategies used for Ag-specific T cell subsets are shown in Supplemental Data Fig. 6.

### scRNA-seq, bulk RNA-seq, and data analysis

Single-cell suspensions isolated from granulomas of Mtb-infected macaques were used for scRNA-seq or bulk RNA-seq studies. scRNA-seq was performed using 10X Genomics Chromium Next GEM Single Cell 3’ Reagent Kits v3.1. Briefly, granuloma cells were stained with unique TotalSeq-A hashtag antibodies [hashtag oligonucleotide (HTO)] (BioLegend) as per the manufacturer’s protocol and equal number of cells from each granuloma were pooled. The pooled cells were stained with propidium iodide (BioLegend) and live cells were sorted. Sorted cells were then super-loaded on a 10X Genomics Next GEM Chip as previously described^29^. Subsequent steps to generate GEX and HTO libraries were performed according to manufacturer’s protocol from 10X Genomics and BioLegend, respectively. The resultant libraries were pooled and sequenced on Illumina NovaSeq 6000 as per 10X Genomics sequencing recommendations.

The sequenced data were aligned to *Macaca mulatta* mmul_10 genome using Cell Ranger (version 6.0.2), then further processed and analyzed using the Seurat (version 4.0) in R (version 4.1.0). Cells were filtered for mitochondrial contamination and low quality, and only singlets, as determined by the HTOs, were included for downstream analysis. Cells from all granulomas were merged and integrated using Harmony. FindAllMarkers with a filter of log fold change ≥0.25 and percent of cells expressing the marker ≥0.25 was used to identify gene markers that distinguish the cell clusters, and the clusters were manually assigned cell types on the basis of identified canonical markers. For the T cell clusters only, an additional quality control filter was used. T cells from both the control and treated groups contained equal number of low quality T cells expressing exceeding low levels of ribosomal genes. A module score containing the genes: RPL3–10, 12–13A, 15, 17–19, 22, 22L1, 23–24, 27–28, 30–32, 34–36, 36AL, 37A–39, 4–5, 6–7A, 8–9, P1–P2, RPS2–5, 7–9, 11–13, 14–18, 20–21, 23–25, 26–27, 27A, 28–29, A, 19BP1, 27L was created. T cells expressing exceedingly low levels of ribosomal genes were not included in downstream analyses. FindMarkers was used to identify DEGs between groups. Genes with a log fold change ≥0.5, percent of cells expressing the marker ≥0.25, and adjusted *P* value ≤ 0.01 were considered significant, and adjusted *P*  ≤  0.05 were considered significant and visualized using feature and violin plots. Gene Ontology enrichment analysis of genes up-regulated at a particular time point was performed using clusterProfiler to identify biological processes (adjusted *p* value ≤ 0.05).

For bulk RNA-seq, total RNA from the granuloma cells was isolated with RNeasy Plus Mini kit (Qiagen) and NHP library preparation was completed with the SMART-Seq v4 Ultra Low Input RNA Kit for Sequencing (Takara Bio Inc.) according to manufacturer’s protocol to create full-length cDNA for each sample. The resultant cDNA was pooled and used to create final sequencing libraries with Nextera XT DNA Library Preparation Kit (Illumina), and the paired-end sequencing was performed on Illumina NovaSeq 6000 system using Illumina NovaSeq S2 flow cells. DESeq2’s was used to identify DEGs between groups and genes with |log2 fold change|> 1 and False Discovery Rate (FDR) < 0.05 were considered as DEGs. To identify possible associations between biological pathways and granulomas’ common DEGs, we performed an enrichment analysis using both gene sets. First, both gene set’s Entrez IDs were retrieved using the clusterProfiler’s bitrfunction alongside the org.Mmu.eg.db database. The enrichment analysis was performed using both gene sets’ fold changes and Entrez IDs as input (*p* value ≤ 0.05).

### IHC and image analysis

Resected granulomas were fixed in 10% neutral-buffered formalin for 16 h, transferred to 70% ethanol and stored at room temperature. Granuloma sections were cut 7-µm thick and dewaxed using xylene/ethanol and then treated with AR6 buffer (Akoya Biosciences) for 20 min at 100 °C. After blocking, tissues were incubated with primary antibodies against IFNγ (clone IFNG/466, 1:400 dilution; Abcam), CD68 (clone KP1, 1:500 dilution; Abcam), CD20 (clone L26, 1:400 dilution; DAKO), NOS2 (polyclonal, 1:100 dilution; Epredia), or IDO (polyclonal, 1:200 dilution; Millipore-Sigma). After washing, slides were stained according to the protocol for the ImmPRESS Duet Double Staining Polymer Kit (Vector Laboratories) and counterstained with hematoxylin. Slides were imaged using Aperio VERSA (Leica Microsystems) and analyzed using QuPath (University of Edinburgh).

To quantify the distribution of B cells in granulomas, scanned images of CD20 chromogenic IHC slides were inported into QuPath. The CD20 and hematoxylin channels were deconvoluted and exported as individual TIFF images. Cell segmentation was performed using both channels with Cellpose3^76^. Following segmentation, B cells were annotated as CD20^+^ segmented cells. The fraction of CD20^+^ B cells was determined relative to the total number of segmented cells per image. To quantify the change in B cell density, the mean distance between each B cell to its 25 nearest B cell neighbors was quantified and compared between groups with a low mean indicating denser spatial association between B cells.

### Cytokine/chemokine Luminex assay

Granuloma homogenates were filter-sterilized and analyzed for protein concentrations using the Luminex NHP XL Cytokine Premixed Kit (R&D Systems). Samples were acquired on a MAGPIX with xPONENT software (Luminex Corporation). Soluble marker protein levels were normalized to total protein levels measured by Quant-iT protein Assay Kit (Thermo Fisher Scientific).

### Measurement of bioactive IFNγ levels with reporter cell line

HEK-Blue IFNγ reporter cells (InvivoGen) were plated at 5 × 10^4^ cells per well in 180 µL in a flat-bottom 96-well plate. 20 µL each of granuloma homogenates was added and incubated overnight at 37 °C. The following day, 20 µL of supernatants from the overnight cell culture was mixed with 180 µL of the QUANTI-Blue solution (InvivoGen) in a flat-bottom 96-well plate. The mixture was incubated at 37 °C for 3 h. The amount of bioactive cytokine was determined with a color change reaction and quantified with a spectrophotometer at 650 nm^29^.

### Measurement of Mtb-specific IgG titer

ELISA plates were coated with Mtb whole-cell lysate (strain H37*Rv*, BEI Resources) at 10 µg/ml diluted in PBS for 1 h at 37 °C. The plates were washed and blocked overnight at 4 °C with block buffer (5% milk powder + 4% whey buffer in PBS Tween-20). Plates were then washed, and plasma samples were added at serial 1:3 dilutions starting at a 1:10 dilution with 4% whey buffer and incubated for 1 h at 37 °C. After washing, plates were incubated with goat anti-monkey IgG (H + L)-HRP (Novus Bio) was added at 1:1000 dilution in 4% whey buffer for 1 h at 37 °C. Plates were washed and 1-Step Ultra-TMB ELISA Substrate Solution (Thermo Scientific) was added to develop the plates. The reaction was stopped by adding 0.5 M sulfuric acid, and the OD measured at 450 nm. The area under the curve of log-transformed values was determined in GraphPad Prism in order to calculate Mtb-specific IgG levels.

### Statistical analysis

All statistical analyses were conducted using GraphPad Prism v10 (GraphPad Software), FlowJo v10 (FlowJo LLC), QuPath, R (The R Foundation), or Python (The Python Foundation). For group comparisons, individual tests vary and are denoted in each figure legend. Multiplex Luminex assay data were log_10_-transformed and normalized by mg of protein and limit of detection values were subtracted. Data were compared between control IgG and rmIFNγR1-IgG groups using the Mann-Whitney *U* test (two-group comparisons) to identify the biomarkers that were statistically significant between two groups. *p* values were adjusted for multiple measurements using Holm-Bonferroni’s method when appropriate. Hierarchical cluster analyses (Ward’s method), with 100× bootstrap of *z* score-normalized data were used to depict the overall expression profile of indicated soluble factors in the study groups.

To visualize the immune landscape and its relationship with IgG groups, the datasets were merged and scaled using z scores. The heatmap was generated with the ComplexHeatmap package, with rows representing immune parameters, and columns representing the treatment groups. Bar plots were included alongside rows to display ford change of each immune parameter, between rmIFNγR1-IgG and the control, with bars colored red for FDR-adjusted *p* values below 0.05 and black for non-significant correlations. The heatmap used a color gradient from blue (lower expression) to yellow (higher expression), and both rows and columns were clustered using Manhattan distance.

sCCA modeling was used to assess whether combinations of soluble factors could discriminate between two groups (R script: www.jstatsoft.org/article/view/v023i12)^52^. To evaluate group separation and identify the most discriminative variables between patient clusters, we performed a Canonical Discriminant Analysis (CDA) using the candisc package in R. The input dataset included predefined IgG groups as the grouping variable and cytokine features as predictors. Prior to model fitting, we assessed multicollinearity among the predictor variables by computing the correlation matrix and applying a cutoff of 0.80 using the findCorrelation function from the caret package. Highly collinear variables were excluded to enhance model stability and interpretability. The CDA was implemented by fitting a multivariate linear model with the retained predictors regressed on the outcome variable, followed by the extraction of canonical dimensions using the candisc function. This approach identifies linear combinations of the predictors (canonical variates) that maximize the separation between the predefined groups. To visualize the results, we projected the individual observations onto the first two canonical dimensions (Canonical coefficient scores) and plotted the scores using scatterplots with 95% confidence ellipses for each cluster. Additionally, bar plots were generated to display the standardized canonical coefficients of each variable in Canonical coefficient scores 1 and 2 separately, facilitating interpretation of the most influential variables contributing to group discrimination.

To infer robust associations among immunological variables, we performed a bootstrapped correlation-based network analysis. Spearman correlation coefficients were computed across 100 bootstrap replicates (sampling with replacement), with statistical significance set at FDR-adjusted *p* value < 0.05. Correlation coefficients were thresholded at |*r*| ≥ 0.4 to retain only moderate to strong associations. For each bootstrap iteration, binary adjacency matrices were created to indicate the presence or absence of significant correlations. These matrices were aggregated to quantify the frequency of each correlation across replicates. Only variable pairs that surpassed a frequency threshold (present in >80% of bootstrap iterations) were retained. Positive and negative correlations were processed separately and then merged to construct a final undirected network. Self-loops and duplicate entries were excluded. Graph construction was performed using the igraph R package. Edge width was scaled according to the bootstrap frequency of correlations, and color intensity was mapped to the correlation strength. Node size was determined by graph strength (sum of edge weights), and nodes were categorized based on predefined biological classes (e.g., cytokines, cell populations, Mtb CFU), each assigned a unique color. Networks were visualized using circular layouts. To further assess network topology, we computed degree centrality and edge density for both positive and negative subnetworks across bootstrap replicates. Chord diagrams were constructed using the circlize R package to provide a circular summary of inter-variable relationships, with edges colored by bootstrap stability. Finally, network metrics, including node degree and global density, were used in subsequent integrative analysis.

### Reporting summary

Further information on research design is available in the Nature Portfolio Reporting Summary linked to this article.

## Supplementary information

Supplementary Information

Reporting summary

Transparent Peer Review file

## Source data

source data

## Data Availability

All RNA sequencing data used in this study are available through the National Center for Biotechnology Information’s Gene Expression Omnibus (GEO) repository at https://www.ncbi.nlm.nih.gov/geo/query/acc.cgi under accession numbers GSE303792 (scRNA-seq from short-term blockade study) and GSE303154 (bulk RNA-seq from long-term blockade study). All other data are in the manuscript and available in the Source file. Source data are provided with this paper.
