# Supplementary material for: IFNγ blockade in Mycobacterium tuberculosis infected macaques alters the granuloma environment but not bacterial control
Source: Nat Commun. 2026 Apr 25;17:5720. doi: 10.1038/s41467-026-72421-9 (PMC13323717; doi:10.1038/s41467-026-72421-9)
Supplement: Supplementary file 1 — Supplementary Information [file 41467_2026_72421_MOESM1_ESM.pdf]

Supplementary Information

IFNγ blockade in *Mycobacterium tuberculosis* infected macaques alters the granuloma environment but not bacterial control

Sakai et al. 2026

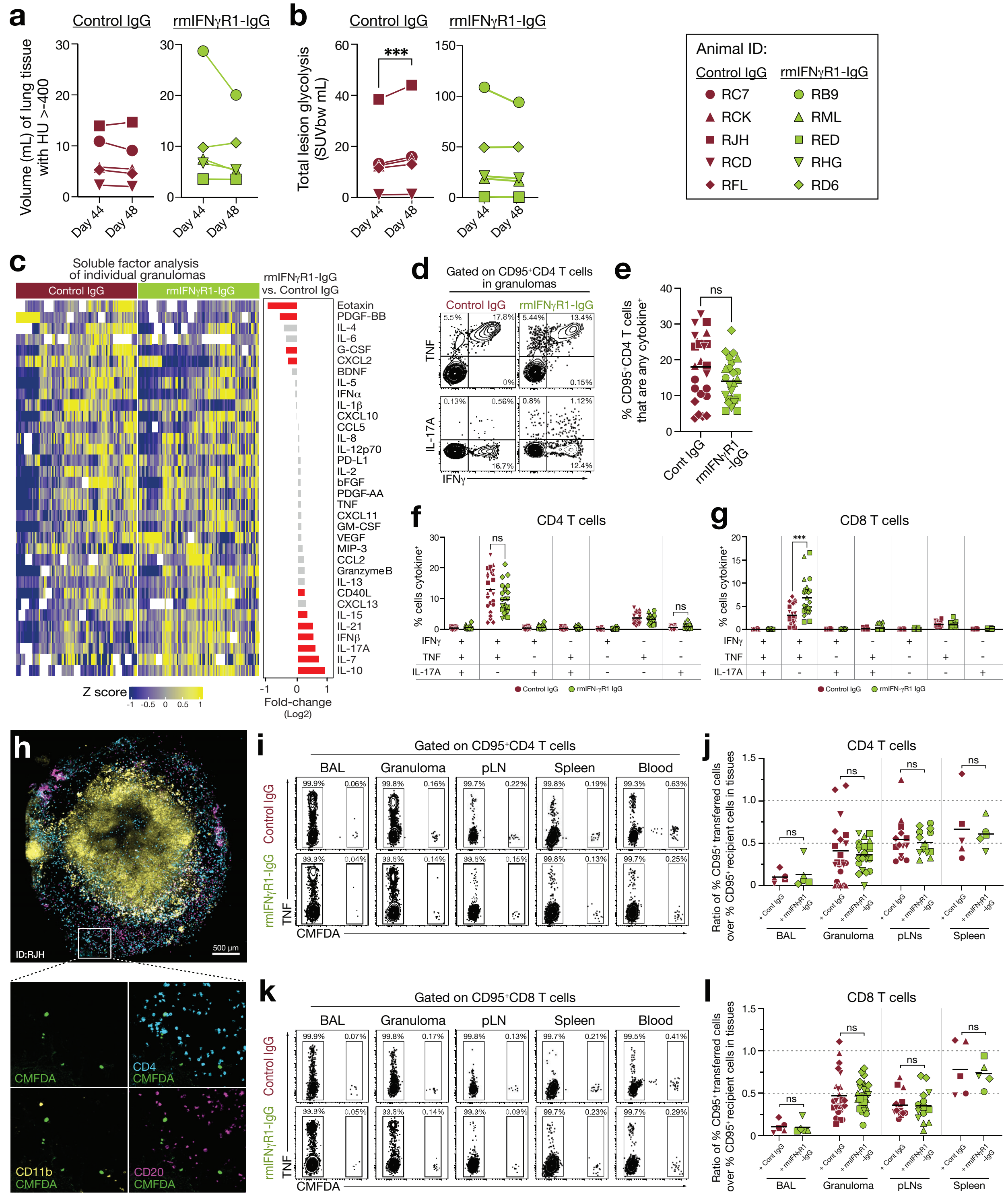

Supplemental Data Fig. 1 | Radiology and immunological characterization of Mtb-infected macaques. a, CT scan analysis of lung lesion size (mL) with >-400 Hounsfield units

(HU) in each animal on day 44 or 48 pi. **b**, Quantification of ^{18}FDG uptake in standard uptake values normalized by body weight (SUVbw) in each animal on day 44 or 48 pi. **c**, Heatmap of soluble factor levels in individual granulomas. Each value was log-transformed and z-score normalized (*left*). Average fold-difference values that reached statistical significance with the Mann-Whitney *U* test adjusted for multiple comparison are shown in red (adjusted $p < 0.05$) (*right*). **d**,**e**, Example FACS plots of cytokine staining on CD95$^+$ CD4 T cells after stimulation with Mtb peptide pools (**d**) and the frequency of Ag-specific CD4 T cells that are either IFNγ$^+$, TNF$^+$ or IL-17A$^+$ (**e**) in granulomas. **f**,**g**, The frequency of CD4 T cells (**f**) and CD8 T cells (**g**) expressing IFNγ$^+$, TNF$^+$ or IL-17A$^+$ in granulomas. **h**, Example image of a granuloma showing the presence of autologously transferred PBMCs (green). Agarose-embedded fresh granulomas were thick-sectioned and stained for CD4, CD11b and CD20. **i-l**, Quantification of autologously transferred cells in each tissue compartment. Example FACS plots of CD4 T cells (**i**) or CD8T cells (**k**) and summary graph showing the ratio of transferred vs. recipient CD4 T cells (**j**) or CD8T cells (**l**) in tissues. ns, non-significant, *** $p < 0.001$ by Student's t test.

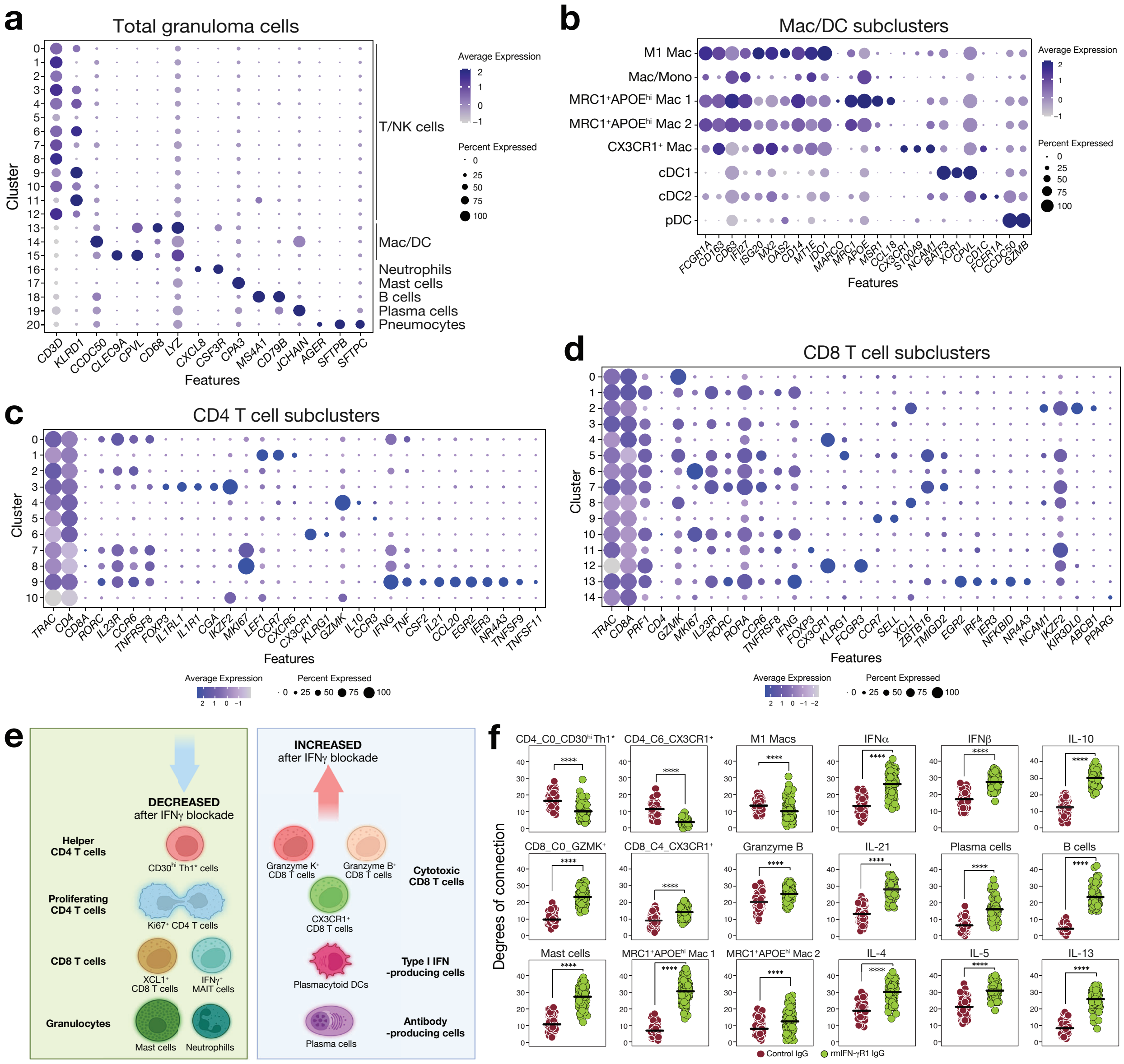

Supplemental Data Fig. 2 | Clustering and network connectivity of individual granuloma cells. a-d, Dot plots showing the expression of cluster-defining genes for total granuloma cells (**a**) and subclusters for Mac/DC (**b**), CD4 T cells (**c**), and CD8 T cells (**d**) in granulomas. Dot size and color intensity represents the percentage and the level of expression, respectively. **e**, Graphical summary of the changes in cellular compositions of granulomas after IFNγ blockade. Created in BioRender. Barber, D. (2026) https://BioRender.com/wb056od. (2025) **f**, The degrees of network connectivity of each variable in 100 bootstrap replicates. **** $p < 0.0001$ by Wilcoxon test.

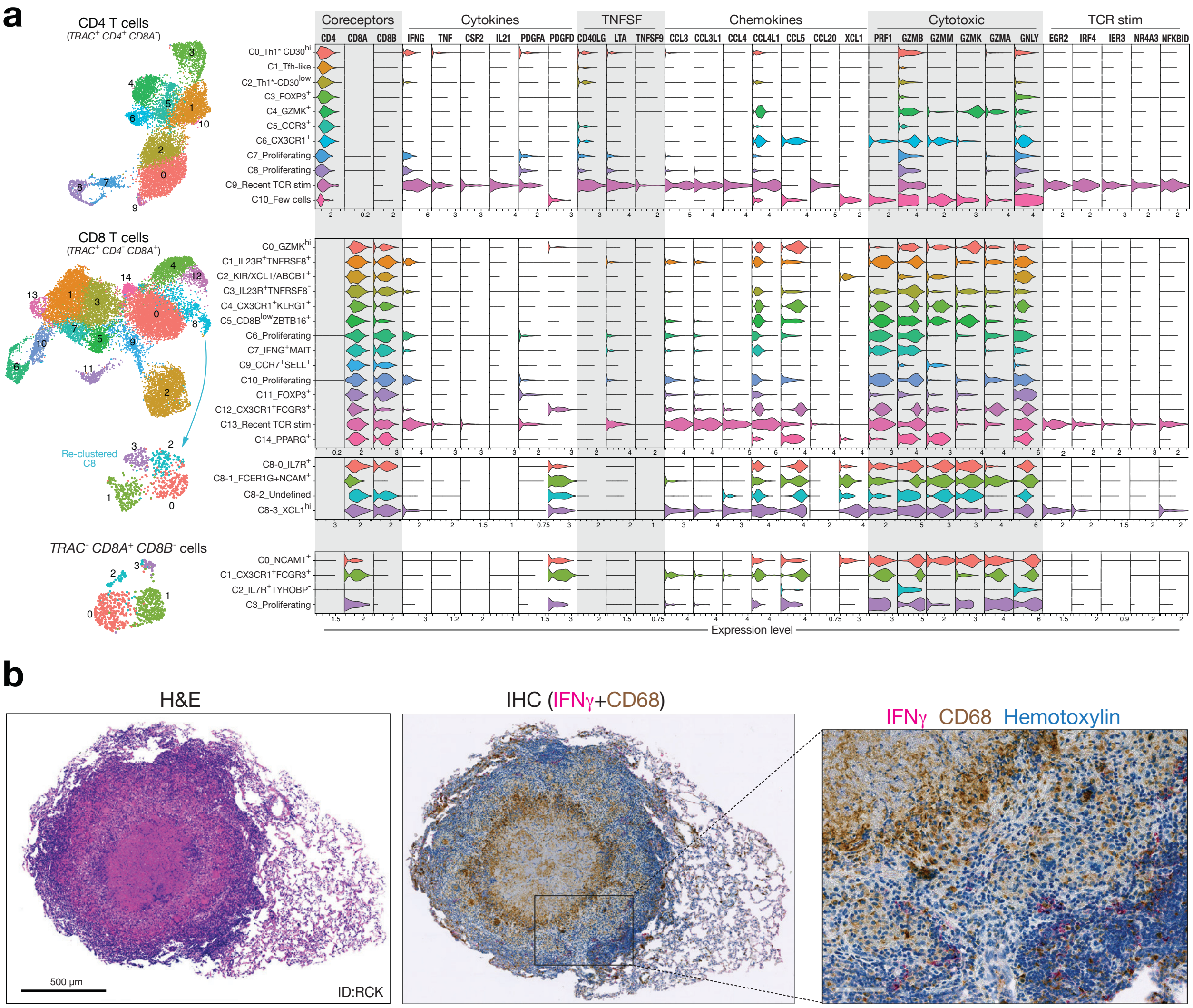

Supplemental Data Fig. 3 | Expression of IFNγ and changes in gene macrophage/DC gene expression after IFNγ blockade. a, Expression of selected effector and recent TCR stimulation genes in T cell subclusters. **b**, Representative H&E staining and IHC of granulomas for IFNγ (magenta) and CD68 (brown).

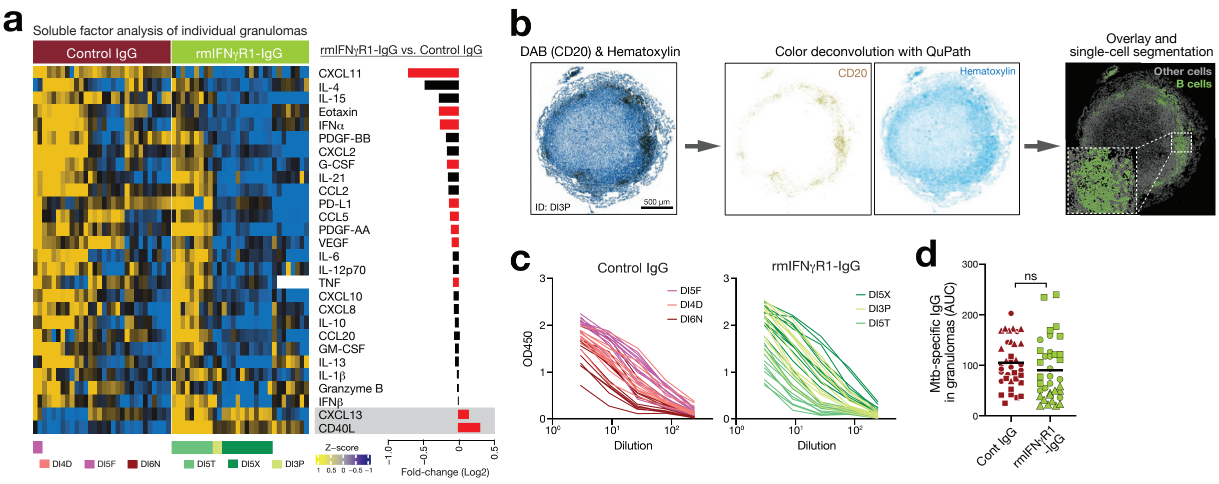

Supplemental Data Fig. 4 | Characterization of inflammatory milieu and B cell responses in granulomas. a, Heatmap of soluble factor levels in individual granulomas (n=10 per animal). Each value was log-transformed and z-score normalized (*left*). Average fold-difference values that reached statistical significance with the Mann-Whitney *U* test adjusted for multiple comparison are shown in red (adjusted $p < 0.05$) (*right*). **b**, Workflow for and image processing using Deepcell-based segmentation of single cells. See also Method section. **c**,**d**, Mtb-specific antibody response at necropsy. Optical density at 450 nm (OD450) for 3-fold serial dilution against Mtb-H37*Rv* lysate of granuloma homogenates (n=12 per animal) (**c**). The area under the curve (AUC) of log-transformed values was determined to calculate Mtb-specific IgG titers (**d**). ns, non-significant by Student's t test.

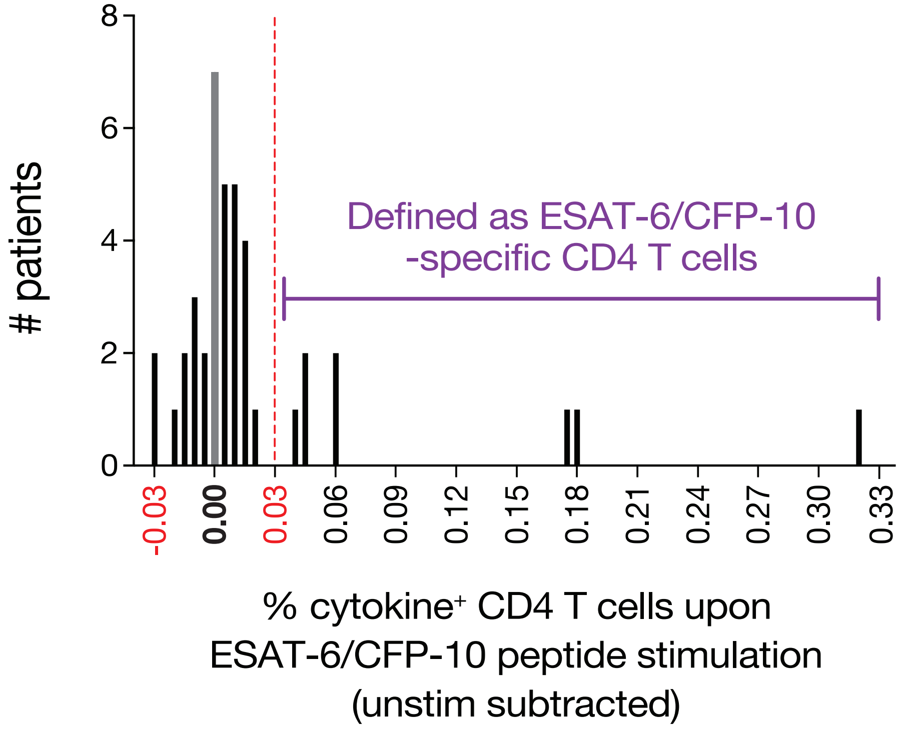

Supplemental Data Fig. 5 | Frequency distribution of ESAT-6/CFP-10–specific CD4 T cells in NTM patients with anti-IFNγ autoAbs. Dashed line indicates an arbitrary threshold (the value from 0 with same distance between 0 and the bottom value [-0.03]) to define the presence of ESAT-6/CFP-10 specific CD4 T cells.

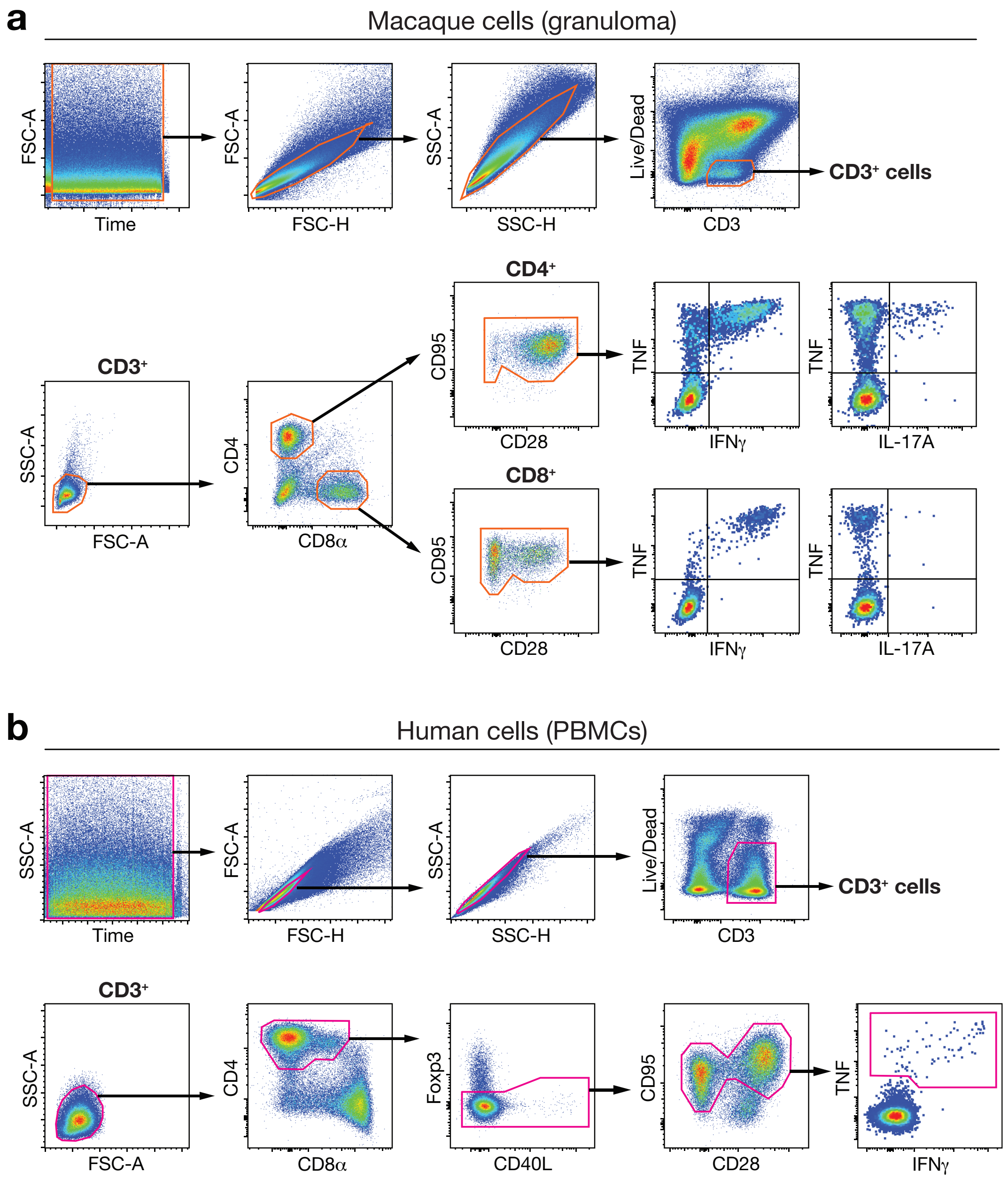

Supplemental Fig. 6 | Gating strategy for Ag-specific T cell analysis by flow cytometry. **a**,**b**, A representative gating strategy for flow cytometric analysis of macaque (**a**) or human (**b**) T cells after in vitro stimulation with antigen peptide pools for 6 hours.

Supplementary Table 1. Clinical characteristics of subjects in this study

			anti-IFNg autoAb patients			
		**Healthy control**	**TB**	***M.kansasii***	**NTM (Cohort 1)**	**NTM (Cohort 2)**
		n=3	n=4	n=1	n=20	n=19
**Sex**	Female	1	3	1	17	17
	(% Female)	(33%)	(75%)	(100%)	(85%)	(89%)
**Age - yrs**	Median	35	43	60	49	50
	(Range)	(28 - 41)	(21 - 55)	-	(19 - 74)	(30 - 74)
**Ethnicity**	Asian	1	4	1	20	19
	(% Asian)	(33%)	(100%)	(100%)	(100%)	(100%)

Supplementary Table 2. List of flow cytometry panels and antibodies used in this study

	Antibody/Dye	Clone	Fluorochrome	Product Info
**Panel 1 for Macaque cells**	TNF	Mab11	BUV395	BD Bioscience, Cat # 563996
	CD4	SK3	BUV496	BD Bioscience, Cat # 612936
	CD95	DX2	BUV737	BD Bioscience, Cat # 612790
	CD3	SP34-2	BUV805	BD Bioscience, Cat # 568354
	Granzyme B	GB11	BV421	BD Bioscience, Cat # 563389
	CD8a	RPA-T8	BV510	BioLegend, Cat # 301036
	IFNg	4S.B3	BV711	BioLegend, Cat # 502540
	(CMFDA)		FITC	Invitrogen, Cat # C7025
	CD28	CD28.8	PE-Dazzle594	BioLegend, Cat # 302942
	IL-17A	eBIO54DEC17	PE-Cy7	Invitrogen, Cat # 25-7179-42
	Live/Dead dye	N/A	Fixable Viability Dye eFlour 780	Invitrogen, Cat # 65-0865-14

	Antibody/Dye	Clone	Fluorochrome	Product Info
**Panel 2 for human cells**	IFNγ	B27	BUV395	BD Bioscience, Cat # 563563
	CD4	SK3	BUV496	BD Bioscience, Cat # 612938
	CD28	CD28.8	BUV737	BD Bioscience, Cat # 612815
	CD8a	SK1	BUV805	BD Bioscience, Cat # 612889
	TNF	Mab11	BV421	BioLegend, Cat # 502932
	CD40L	24-31	BV605	BioLegend, Cat # 310826
	CD95	DX2	BV711	BioLegend, Cat # 305644
	CD3	SP34-2	BV786	BD Bioscience, Cat # 563918
	IL-17A	eBIO64DEC17	PECy7	Invitrogen, Cat # 25-7179-42
	Foxp3	PCH101	AF700	Invitrogen, Cat # 56-4776-41
	Live/Dead dye	N/A	Fixable Viability Dye eFlour 780	Invitrogen, Cat # 65-0865-14
