# Supplementary material for: IFNγ blockade in Mycobacterium tuberculosis infected macaques alters the granuloma environment but not bacterial control
Source: Nat Commun. 2026 Apr 25;17:5720. doi: 10.1038/s41467-026-72421-9 (PMC13323717; doi:10.1038/s41467-026-72421-9)
Supplement: Supplementary file 2 — Reporting summary [file 41467_2026_72421_MOESM2_ESM.pdf]

nature portfolio

Corresponding author(s): Daniel Barber

Last updated by author(s): Apr 1, 2026

Reporting Summary

Nature Portfolio wishes to improve the reproducibility of the work that we publish. This form provides structure for consistency and transparency in reporting. For further information on Nature Portfolio policies, see our Editorial Policies and the Editorial Policy Checklist.

Statistics

For all statistical analyses, confirm that the following items are present in the figure legend, table legend, main text, or Methods section.

n/a	Confirmed	
☐	☒	The exact sample size (*n*) for each experimental group/condition, given as a discrete number and unit of measurement
☐	☒	A statement on whether measurements were taken from distinct samples or whether the same sample was measured repeatedly
☐	☒	The statistical test(s) used AND whether they are one- or two-sided<br>*Only common tests should be described solely by name; describe more complex techniques in the Methods section.*
☐	☒	A description of all covariates tested
☐	☒	A description of any assumptions or corrections, such as tests of normality and adjustment for multiple comparisons
☐	☒	A full description of the statistical parameters including central tendency (e.g. means) or other basic estimates (e.g. regression coefficient) AND variation (e.g. standard deviation) or associated estimates of uncertainty (e.g. confidence intervals)
☐	☒	For null hypothesis testing, the test statistic (e.g. *F*, *t*, *r*) with confidence intervals, effect sizes, degrees of freedom and *P* value noted<br>*Give P values as exact values whenever suitable.*
☒	☐	For Bayesian analysis, information on the choice of priors and Markov chain Monte Carlo settings
☒	☐	For hierarchical and complex designs, identification of the appropriate level for tests and full reporting of outcomes
☐	☒	Estimates of effect sizes (e.g. Cohen's *d*, Pearson's *r*), indicating how they were calculated

Our web collection on statistics for biologists contains articles on many of the points above.

Software and code

Policy information about availability of computer code

Data collection	FACS DIVA, Nucline, MAGPIX xPONENT, Aperio ImageScope
Data analysis	FlowJo, MIM Maestro, Osirix v.5.9, QuPath, Cellpose, Python, R, circlize R, igraph R, and Graphpad Prism

For manuscripts utilizing custom algorithms or software that are central to the research but not yet described in published literature, software must be made available to editors and reviewers. We strongly encourage code deposition in a community repository (e.g. GitHub). See the Nature Portfolio guidelines for submitting code & software for further information.

Data

Policy information about availability of data

All manuscripts must include a data availability statement. This statement should provide the following information, where applicable:

- Accession codes, unique identifiers, or web links for publicly available datasets
- A description of any restrictions on data availability
- For clinical datasets or third party data, please ensure that the statement adheres to our policy

All RNA sequencing data used in this study are available through the National Center for Biotechnology Information's Gene Expression Omnibus (GEO) repository at https://www.ncbi.nlm.nih.gov/geo/query/acc.cgi under accession numbers GSE303792 (scRNA-seq from short term blockade study) and GSE303154 (bulk RNA-seq from long term blockade study). All other data are in the manuscript and available in the Source file.

Research involving human participants, their data, or biological material

Policy information about studies with human participants or human data. See also policy information about sex, gender (identity/presentation), and sexual orientation and race, ethnicity and racism.

Reporting on sex and gender	The clinical characteristics of subjects in this study was provided in the Suppl. Table 1.
Reporting on race, ethnicity, or other socially relevant groupings	Ethnicity: healthy control group (n=3) - one Black, one White and one Asian ancestryparticipant. anti-IFNg autoAb group (n=44) - all Asian participants.
Population characteristics	The population characteristics can be seen in the Suppl. Table 1.
Recruitment	Participant recruitment was conducted based on IRB-approved protocols listed in the Method section.
Ethics oversight	Human studies were conducted under Institutional Review Board-approved protocols NCT00814827, NCT00018044, NCT00001355, and NCT01212003. Written informed consent was obtained from all participants in the human cohorts. Healthy control whole blood samples were obtained from anonymous donors through the NIH Blood Bank in accordance with institutional policies.

Note that full information on the approval of the study protocol must also be provided in the manuscript.

Field-specific reporting

Please select the one below that is the best fit for your research. If you are not sure, read the appropriate sections before making your selection.

☒ Life sciences ☐ Behavioural & social sciences ☐ Ecological, evolutionary & environmental sciences

For a reference copy of the document with all sections, see nature.com/documents/nr-reporting-summary-flat.pdf

Life sciences study design

All studies must disclose on these points even when the disclosure is negative.

Sample size	No power calculations were performed to pre-determine sample sizes.
Data exclusions	No data was excluded from the analysis.
Replication	In mouse experiments, all experiments were independently repeated at least twice with biological replicates. NHP experiments are done once each.
Randomization	No randomization was done.
Blinding	No blinding was performed.

Reporting for specific materials, systems and methods

We require information from authors about some types of materials, experimental systems and methods used in many studies. Here, indicate whether each material, system or method listed is relevant to your study. If you are not sure if a list item applies to your research, read the appropriate section before selecting a response.

Materials & experimental systems

n/a	Involved in the study	
☐	☒	Antibodies
☐	☒	Eukaryotic cell lines
☒	☐	Palaeontology and archaeology
☐	☒	Animals and other organisms
☒	☐	Clinical data
☒	☐	Dual use research of concern
☒	☐	Plants

Methods

n/a	Involved in the study	
☒	☐	ChIP-seq
☐	☒	Flow cytometry
☒	☐	MRI-based neuroimaging

Antibodies

Antibodies used	The antibody information usd in this study was provided in the Suppl. Table 2.
Validation	All antibodies were purchased from commercial suppliers and antibody titers were validated prior to the experiments.

Eukaryotic cell lines

Policy information about cell lines and Sex and Gender in Research

Cell line source(s)	HEK-Blue IFNγ reporter cells (InvivoGen)
Authentication	N/A
Mycoplasma contamination	The cell line was tested negative for mycoplasma contamination.
Commonly misidentified lines (See ICLAC register)	No commonly misidentified cell lines were used.

Animals and other research organisms

Policy information about studies involving animals; ARRIVE guidelines recommended for reporting animal research, and Sex and Gender in Research

Laboratory animals	Rhesus macaques and C57BL/6, ifng-/-, nos2-/-, huNOS2+/0mNOS2-/- (hNOS2-Tg), ido1-/-ido2-/- (IDO1/2dKO)mice.
Wild animals	N/A
Reporting on sex	All male macaques were used in this study. Both female and male mice were used in the study.
Field-collected samples	N/A
Ethics oversight	All animal procedures were approved by the National Institute of Allergy and Infectious Diseases (NIAID) Division of Intramural Research Animal Care and Use Committee (ACUC) under study proposal LPD-25E and were performed in accordance with the Animal Welfare Act, the Guide for the Care and Use of Laboratory Animals, and all applicable institutional regulations, standards, and policies.

Note that full information on the approval of the study protocol must also be provided in the manuscript.

Plants

Seed stocks	*Report on the source of all seed stocks or other plant material used. If applicable, state the seed stock centre and catalogue number. If plant specimens were collected from the field, describe the collection location, date and sampling procedures.*
Novel plant genotypes	*Describe the methods by which all novel plant genotypes were produced. This includes those generated by transgenic approaches, gene editing, chemical/radiation-based mutagenesis and hybridization. For transgenic lines, describe the transformation method, the number of independent lines analyzed and the generation upon which experiments were performed. For gene-edited lines, describe the editor used, the endogenous sequence targeted for editing, the targeting guide RNA sequence (if applicable) and how the editor was applied.*
Authentication	*Describe any authentication procedures for each seed stock used or novel genotype generated. Describe any experiments used to assess the effect of a mutation and, where applicable, how potential secondary effects (e.g. second site T-DNA insertions, mosiacism, off-target gene editing) were examined.*

Flow Cytometry

Plots

Confirm that:

☒ The axis labels state the marker and fluorochrome used (e.g. CD4-FITC).

☒ The axis scales are clearly visible. Include numbers along axes only for bottom left plot of group (a 'group' is an analysis of identical markers).

☒ All plots are contour plots with outliers or pseudocolor plots.

☒ A numerical value for number of cells or percentage (with statistics) is provided.

Methodology

Sample preparation	Detailed protocol of cell preparation and staining was provided in the Method section.
Instrument	BD FACS Symphony A5 and BD FACS Aria III
Software	FlowJo 10
Cell population abundance	Live Dead stain-negative live cell population (2 to 98% of total cells depending on their tissue origin) was subjected to analysis.
Gating strategy	Gating strategies can be seen in Suppl. Fig. 6.

☒ Tick this box to confirm that a figure exemplifying the gating strategy is provided in the Supplementary Information.
