# Supplementary material for: IFNγ blockade in Mycobacterium tuberculosis infected macaques alters the granuloma environment but not bacterial control
Source: Nat Commun. 2026 Apr 25;17:5720. doi: 10.1038/s41467-026-72421-9 (PMC13323717; doi:10.1038/s41467-026-72421-9)
Supplement: Supplementary file 3 — Transparent Peer Review file [file 41467_2026_72421_MOESM3_ESM.pdf]

nature portfolio

Peer Review File

IFNγ blockade in *Mycobacterium tuberculosis* infected macaques alters the granuloma environment but not bacterial control

Corresponding Author: Dr Daniel Barber

This file contains all reviewer reports in order by version, followed by all author rebuttals in order by version.

Version 0:

Reviewer comments:

Reviewer #1

(Remarks to the Author)
Thank you for a detailed rebuttal document. The edits/revision have addressed questions from the prior review.

Reviewer #3

(Remarks to the Author)
In this paper, an excellent group of investigators reinvestigate the role of IFN in protection against tuberculosis. Their work reexamines the role of IFN in control of tuberculosis, and they found that IFN is less important in disease control as previously thought. To prove this point, investigators inhibited IFN in different phases of Mtb infection in primates and humanized INOS promoter in mice as well as examining different mycobacterial infections in humans displaying anti-IFN antibodies. The data presented are high quality, informative, but it does not exclude a role for IFN to control tuberculosis.
1. The paper largely focuses on the pathophysiology of an IFN trap, which captures around 80% of IFN available, in a NHP system. Researchers characterized two treatment regiments – the first treatment schedule was a longer-term treatment that began at the start of infection, and the second treatment schedule was a short-term inhibition that occurred after granuloma formation. The researchers provided an outstanding data set consisting of multiple modalities including several imaging modalities, flow cytometry, and cytokine ELISA assays in addition to transcriptomic data. The results are well discussed. Parameters measured included granuloma size, cellular composition, cytokine production, and metabolic changes, which do require high IFN . The description of broad changes induced by high level of IFN during the course of mycobacterial host response is excellent and provides a great resource for the field. Bacterial load was affected, but that is not proof against a role of IFN in protection as 20% IFN may be sufficient to drive various responses. These data do identify a long list of host responses that are not required on a non-redundant way for protection against mycobacteria, which raises many interest questions in field. As a comment, some of the data hard to interpret and the different methodology in sequencing may restrict the comparison of two different treatments. However, these results contribute a significant progress in the field.
2. The role of INOS is less clear in humans than in mice. However, the results showing that IFN induces less INOS driven by human promoter does not preclude the possibility that IFN does not interfere with protection through alternative pathways. The possibility that INOS is less of a factor is supported through the NHP data, which had an absence of INOS increase in NHP lung granulomas.
3. The patients with anti-IFN represent an interesting cohort. One aspect that could contribute to the response is the prevalence of NTM, is that NTM much more prevalent in comparison to Mtb even in endemic areas. This can be similar to humans with mutations in the in the IFN pathway where NTM is more frequent but Mtb susceptibility is also increased. The adult onset of the disease suggests a scenario that Mtb peptide positives had IFN to protect against infection before anti IFN response was present. It is still interesting that reactivation is not induced by the block of IFN .
In summary: The paper offers 3 different findings summarized above. While they are all of interest, the first point offers the most value to the field in identifying the different high IFN induced events at different phases of an infectious response. One weaker aspect of the paper is the link between the three separate aspects as it does feel somewhat forced.

Open Access This Peer Review File is licensed under a Creative Commons Attribution 4.0 International License, which permits use, sharing, adaptation, distribution and reproduction in any medium or format, as long as you give appropriate credit to the original author(s) and the source, provide a link to the Creative Commons license, and indicate if changes were made.
In cases where reviewers are anonymous, credit should be given to 'Anonymous Referee' and the source.
The images or other third party material in this Peer Review File are included in the article's Creative Commons license, unless indicated otherwise in a credit line to the material. If material is not included in the article's Creative Commons license and your intended use is not permitted by statutory regulation or exceeds the permitted use, you will need to obtain permission directly from the copyright holder.
To view a copy of this license, visit https://creativecommons.org/licenses/by/4.0/

Reviewer #3 (Remarks to the Author):

"In this paper, an excellent group of investigators reinvestigate the role of IFNγ in protection against tuberculosis. Their work reexamines the role of IFNγ in control of tuberculosis, and they found that IFNγ is less important in disease control as previously thought. To prove this point, investigators inhibited IFNγ in different phases of Mtb infection in primates and humanized INOS promoter in mice as well as examining different mycobacterial infections in humans displaying anti- IFNγ antibodies. The data presented are high quality, informative, but it does not exclude a role for IFNγ to control tuberculosis.

1. The paper largely focuses on the pathophysiology of an IFNγ trap, which captures around 80% of IFNγ available, in a NHP system. Researchers characterized two treatment regiments – the first treatment schedule was a longer-term treatment that began at the start of infection, and the second treatment schedule was a short-term inhibition that occurred after granuloma formation. The researchers provided an outstanding data set consisting of multiple modalities including several imaging modalities, flow cytometry, and cytokine ELISA assays in addition to transcriptomic data. The results are well discussed. Parameters measured included granuloma size, cellular composition, cytokine production, and metabolic changes, which do require high IFNγ. The description of broad changes induced by high level of IFNγ during the course of mycobacterial host response is excellent and provides a great resource for the field. Bacterial load was affected, but that is not proof against a role of IFNγ in protection as 20% IFNγ may be sufficient to drive various responses. These data do identify a long list of host responses that are not required on a non-redundant way for protection against mycobacteria, which raises many interest questions in field. As a comment, some of the data hard to interpret and the different methodology in sequencing may restrict the comparison of two different treatments. However, these results contribute a significant progress in the field."

We than the Reviewer for their kind comments. The Reviewer is correct that we cannot rule out any role at all for IFNγ in control of Mtb. However, we will point out that in many granulomas there was a complete block of IFNγ, and it was these granulomas where the bacterial loads were the lowest. Thus, we achieved far greater than 80% blockade in most lesions and there was no impact on bacterial control. Taken with our results showing that people with high levels of anti-IFNγ neutralizing auto Abs can sometimes develop extrapulmonary NTM disease rather than pulmonary TB, despite having been exposed to Mtb, strongly indicates that Mtb can be controlled without a lot of IFNγ. In short, we suggest that while our data do not rule out any role for IFNγ, it clearly makes the case that it is far less important than thought based on mouse model data.

"2. The role of INOS is less clear in humans than in mice. However, the results showing that IFNγ induces less INOS driven by human promoter does not preclude the possibility that IFNγ does not interfere with protection through alternative pathways. The possibility that INOS is less of a factor is supported through the NHP data, which had an absence of INOS increase in NHP lung granulomas."

We appreciate the Reviewer mentioning the iNOS data. During the course of this review, a manuscript by the Corleis lab examining the species specificity of IFNγ driven iNOS was been deposited on bioRxiv (https://www.biorxiv.org/content/10.64898/2026.01.11.697682v1). They screened macrophages from many different species and found that only in mice (and to a lesser extent cattle) does IFNγ drive the induction of iNOS. This preprint strongly supports our conclusions that mice overestimate the importance of IFNγ in control of Mtb due to the unique ability of murine macrophages to produce massive amounts of NO radicals in response to IFNγ.

Moreover, we reanalyzed a newly published human TB granuloma snRNAseq dataset from the Tobin lab (PMID: 40772762). We found that macrophages from human granulomas also do not express iNOS (*NOS2*) but do express IDO1 and IDO2 (Reviewer Figure 1). Thus, the new data from the Corlies preprint and recent Tobin studies support our conclusion that IFNγ-driven iNOS-mediated suppression of Mtb infection is unique to mice.

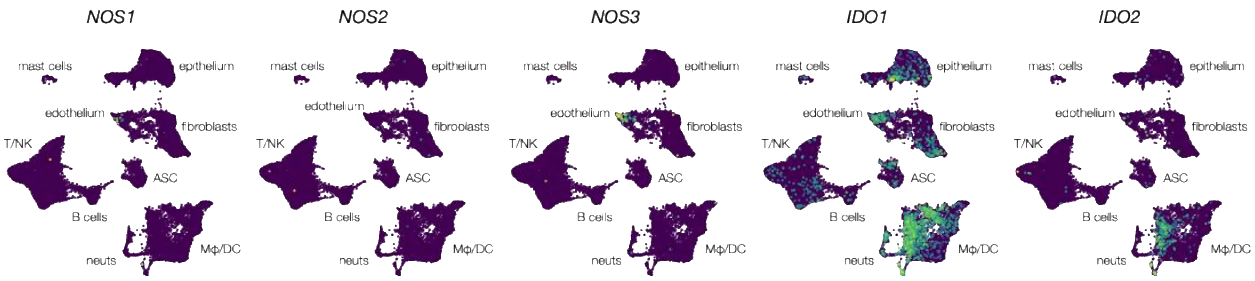

Reviewer Figure #1. NOS and IDO expression in human TB granulomas. Single nucleus RNAseq data from human TB lesions are re-analyzed from PMID: 40772762.

“3. The patients with anti-IFNγ represent an interesting cohort. One aspect that could contribute to the response is the prevalence of NTM, is that NTM much more prevalent in comparison to Mtb even in endemic areas. This can be similar to humans with mutations in the in the IFNγ pathway where NTM is more frequent but Mtb susceptibility is also increased. The adult onset of the disease suggests a scenario that Mtb peptide positives had IFNγ to protect against infection before anti IFNγ response was present. It is still interesting that reactivation is not induced by the block of IFNγ. In summary: The paper offers 3 different findings summarized above. While they are all of interest, the first point offers the most value to the field in identifying the different high IFNγ induced events at different phases of an infectious response. One weaker aspect of the paper is the link between the three separate aspects as it does feel somewhat forced.”

To connect these major parts of the study more clearly, we have moved the iNOS expression data and murine model survival data from the Mtb infected humanized iNOS and *Ido1*$^{-/-}$*Ido2*$^{-/-}$ mice from the supplemental figure to the main text (now in Figure 3).

We have also added new text to more clearly connect these major points in the paper. In short, the demonstration that auto-Ab mediated IFNγ neutralization in humans does not lead to TB in previously Mtb exposed individuals supports our macaque data showing that neutralizing IFNγ doesn't exacerbate TB. The decades old dogma that IFNγ is the primary mediator of adaptive immunity to Mtb infection is mostly based on murine model data, so we felt it was critical to explain why mice differ so much from humans and NHP. The major mechanism of IFNγ-mediated control of Mtb infection in mice is through iNOS induction, but we don't find iNOS in NHP or human granulomas. This led us to hypothesize that the special ability of mice to produce nitric oxide in response to IFNγ explains their reliance on IFNγ. Indeed, we found that humanized iNOS mice are highly susceptible to TB. Thus, mice overestimate the importance of IFNγ because of a highly protective IFNγ-induced response that doesn't exist in humans and NHP. In other words, mice got it wrong because of iNOS.
